# Activation of Spinal Astrocyte α2A Adrenoceptors Protects Against Sepsis‐Induced Heart Injury Through Inhibition of GABAergic Neuronal Necroptosis

**DOI:** 10.1002/advs.202504406

**Published:** 2025-07-21

**Authors:** Ruilin He, Bin Wu, Liuhu Han, Mingde Li, Wenli Guo, Jiajing Fu, Bin Mei, Eric R. Gross, Xuesheng Liu, Yao Lu

**Affiliations:** ^1^ Department of Anesthesiology The First Affiliated Hospital of Anhui Medical University Key Laboratory of Anesthesia and Perioperative Medicine of Anhui Higher Education Institutes Anhui Medical University Hefei 230022 China; ^2^ Department of Anesthesiology Perioperative and Pain Medicine School of Medicine Stanford University Stanford CA 94305 USA

**Keywords:** andrenergic receptor alpha‐2, astrocyte, GABAergic neurons, sepsis‐induced cardiomyopathy, spinal

## Abstract

The peripheral immune system contributes to the development of sepsis‐induced cardiomyopathy. However, the underlying mechanisms linking central immune cells and neurons to sepsis‐induced cardiomyopathy remain to be clarified. Here, acute sepsis is induced by cecal ligation puncture (CLP), and pharmacological and RNAi interventions are administered to the thoracic spinal cord via intrathecal injection. Echocardiography and histology confirm reduced cardiac function following CLP. Sepsis‐induced spinal cord changes involved neuronal activation and loss with decreased gamma‐aminobutyric acid (GABA) levels. Necroptosis effector genes are markedly upregulated with increased RIPK1, RIPK3, and MLKL co‐expression evident in spinal GABAergic neurons, while administration of the necroptosis inhibitor Necrostatin‐1 substantially preserves neurons and reverses sepsis‐associated cardiac functional changes. Sepsis triggers increased C3, IL‐6 and TNF‐α in spinal astrocytes, while administration of the α2A‐adrenergic receptor (α2‐AR) agonist dexmedetomidine blocked inflammatory factor production, neuronal damage, and cardiac dysfunction. These findings suggest that sepsis‐induced cardiomyopathy arises from a neuroimmune interplay involving spinal astrocyte activation, GABAergic neuronal necroptosis, and cardiac damage driven by sympathetic hyperstimulation.

## Graphical abstract

The study in this paper found that activating the α2A receptors of astrocytes in the thoracic spinal cord can reduce the release of inflammatory factors, thereby decreasing the necroptosis of GABAergic neurons and consequently alleviating myocardial injury caused by sepsis.

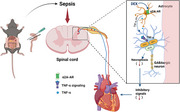

## 1 Introduction

Sepsis, defined as life‐threatening organ dysfunction caused by a dysregulated host response to infection, is responsible for ≈11 million annual deaths worldwide.^[^ ^1^, ^2^ ^]^ A significant proportion of septic patients display aberrant cardiac function, termed sepsis‐induced cardiomyopathy (SICM, see Table S1, Supporting Information for all acronyms in the manuscript) or dysfunction (SIDM), and such patients exhibit significantly worse short‐term mortality. The understanding of SICM remains incomplete, and effective treatments are urgently needed due to its potential to increase short‐term mortality. Intensive research efforts have implicated various factors associated with myocardial oedema^[^ ^3^ ^]^ along with knowledge that the immune system plays a critical role in the progression of sepsis.^[^ ^4^ ^]^ Regarding the latter, research has predominantly focused on the role of inflammatory cytokines and other regulatory mechanisms associated with peripheral immune cells.^[^ ^5^, ^6^, ^7^ ^]^ Although previous studies have shown a link between the vagus nerve and systemic inflammatory response.^[^ ^8^, ^9^ ^]^ Nonetheless, the nexus that exists between immunity and the central nervous system in sepsis is incompletely understood.

The nervous system plays multifaceted roles in heart function regulation.^[^ ^10^, ^11^ ^]^ Gamma‐aminobutyric acid (GABA) is the main inhibitory neurotransmitter in the central nervous system (CNS), and GABAergic neuronal dysfunction occurs in models of sepsis‐associated encephalopathy.^[^ ^12^ ^]^ Moreover, other studies identify that spinal cord stimulation can reduce sympathetic nervous system excitation and ventricular arrhythmia induced by cardiac ischemia‐reperfusion by activating the GABAergic signaling pathway. Indeed, administering small doses of opioid drugs intrathecally to activate spinal cord central opioid receptors can alleviate cardiac ischemia injury, thus highlighting a central role for the spinal cord in mediating myocardial protection. Upregulation of GABAARδ in the thoracic spinal cord can significantly reduce myocardial infarct size, arrhythmia, and myocardial cell apoptosis after cardiac ischemia injury.^[^ ^13^, ^14^, ^15^, ^16^ ^]^ In the early stages of sepsis, there is a marked inflammatory response and overactivation of the sympathetic nervous system.^[^ ^17^ ^]^ Other evidence suggests that sustained sympathetic hyperactivity may impair myocardial contractility and potentially induce cell death.^[^ ^18^ ^]^ In septic patients, elevated blood levels of cardiac troponin T, a marker of acute coronary syndromes, have been associated with left ventricular systolic dysfunction and myocardial injury.^[^ ^19^, ^20^ ^]^ Moreover, it is hypothesized that inflammation fundamentally underlies the leakage of cytosolic components such as troponin T from myocardial cells,^[^ ^21^, ^22^ ^]^ further impairing myocardial contractile function.^[^ ^17^, ^23^ ^]^

Interactions between glia and neurons are critical for maintaining CNS homeostasis and play pivotal roles in responding to infection and injury.^[^ ^24^, ^25^ ^]^ Astrocytes, the most abundant glial cells in the CNS, influence the activity of GABAergic neurons.^[^ ^26^, ^27^, ^28^, ^29^ ^]^ For instance, they respond to spinal cord injury by modulating neuronal activity and exerting analgesic effects.^[^ ^30^, ^31^, ^32^, ^33^, ^34^ ^]^ Recent studies report that astrocytes are involved in CNS‐mediated responses to myocardial injury.^[^ ^35^, ^36^, ^37^ ^]^ Additional findings from mouse models showed that sepsis induces elevated α2A‐adrenergic receptor (α2A‐AR) expression on hippocampal astrocytes, a response which could be effectively inhibited by systemic administration of α2A‐AR agonists.^[^ ^38^ ^]^ Moreover, α2‐adrenergic receptor agonists such as dexmedetomidine (DEX) provide cardiovascular protection and show effectiveness in stabilizing hemodynamics in septic patients.^[^ ^39^, ^40^, ^41^ ^]^ Intriguingly, the actions of DEX on reducing patient mortality have been linked with the suppression of pro‐inflammatory cytokine responses and inhibition of systemic inflammation.^[^ ^42^, ^43^, ^44^, ^45^ ^]^

Different studies have shown that inhibiting sympathetic overactivity or vagus nerve stimulation can suppress and weaken inflammatory responses.^[^ ^8^, ^9^, ^46^ ^]^ Thus, we hypothesized that α2A‐AR stimulation during sepsis would inhibit astrocyte activation in the dorsal horn of the spinal cord. Furthermore, the ensuing effects on GABAergic neurons would therefore alleviate sympathetic overexcitation and mitigate sepsis‐induced myocardial injury and functional decline.

## 2 Results

### 2.1 Sepsis Induces Significant Myocardial Damage

**Figure 1:**
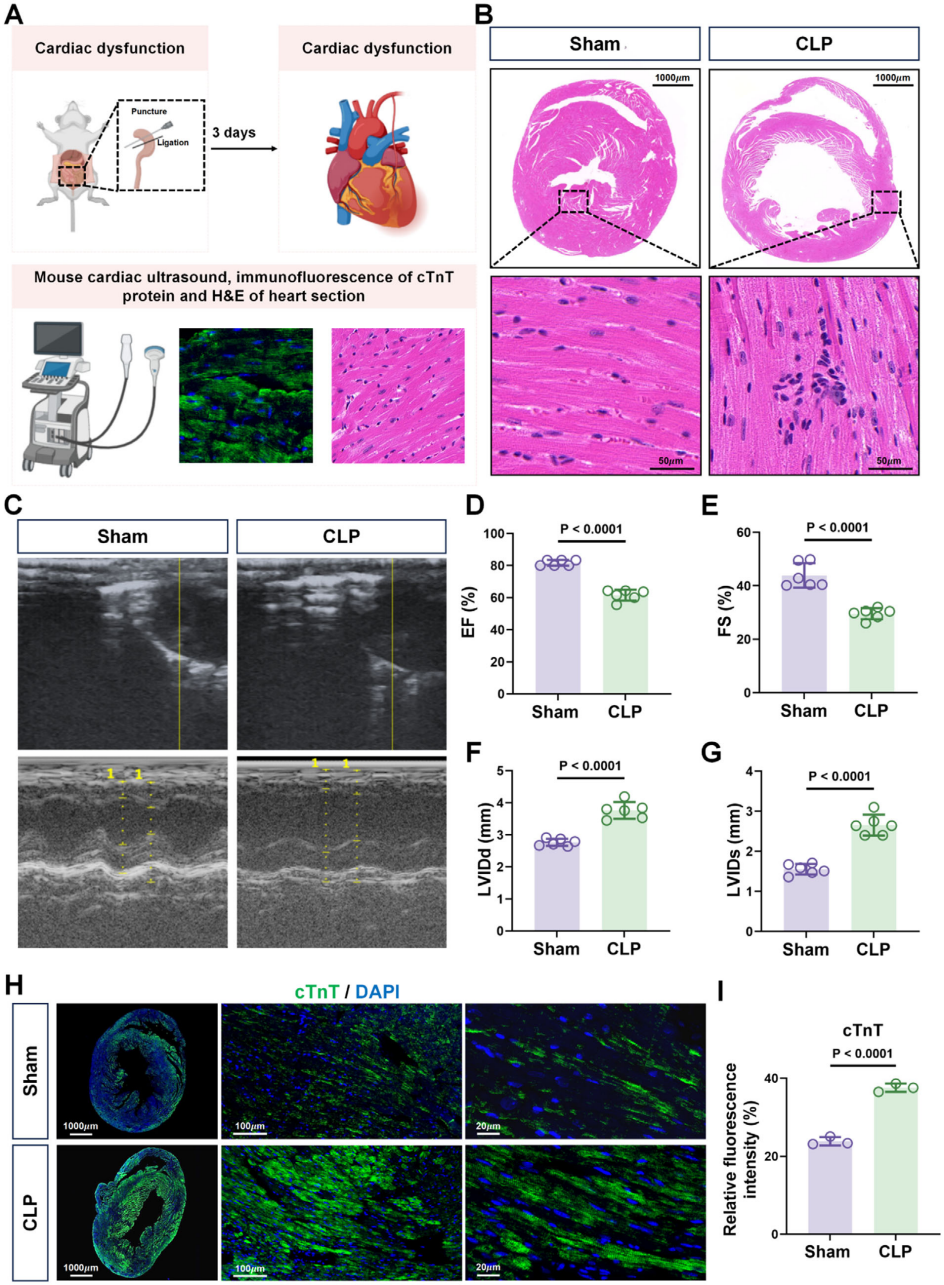
Sepsis caused by cecal ligation and perforation leads to myocardial damage and reduced cardiac function. A) Schematic of the experimental procedure. B) Representative images of hematoxylin and eosin‐stained heart sections from Sham or CLP operated mice three days after the procedure. Scale bars: 1000 µm, 20 µm. C–G) Representative echocardiograms of Sham and CLP mice (C), with plots summarizing cardiac ejection fraction (EF; D), fractional shortening (FS; E), left ventricular end‐diastolic internal diameter (LVIDd; F), and end‐systolic internal diameter (LVIDs; G) in Sham and CLP mice (n = 6 mice/group). H,I) Representative epifluorescence images of immunostaining against cTnT (green) in heart sections from Sham and CLP mice. Nuclei were counterstained with DAPI (blue) throughout the immunostaining experiments. Scale bars: 1000, 100, 20 µm (H). Relative cTnT levels in (H) were estimated from fluorescence intensity measurements (n = 3/ sections group) (I). Data are shown as mean ± SD; two‐tailed Student's *t*‐test.

After cecal ligation and perforation (CLP), myocardial damage was evaluated three days post‐CLP using echocardiography combined with staining of myocardial tissue using hematoxylin and eosin (HE), together with immunofluorescence staining against cardiac troponin T (cTnT) (**Figure** **1**A). Compared to the sham group, histological analysis revealed significant increases in inflammatory cell infiltration into the heart after CLP (Figure 1B). Consistently, there were notable increases in cTnT expression in the CLP group compared to the sham group (Figure 1H–I). Moreover, echocardiographic analysis demonstrated that CLP group mice exhibited marked reductions in ejection fraction (EF) and fractional shortening (FS), alongside a significant enlargement of the left ventricular internal diameter at end‐diastole (LVIDd) and end‐systole (LVIDs) (Figure 1C–G). Together, these results demonstrate that sepsis induces significant myocardial injury and impairs cardiac function in an acute mouse model of sepsis.

### 2.2 Activation of Thoracic Spinal Cord Astrocytes and Neurons in Sepsis‐Induced Myocardial Injury

**Figure 2:**
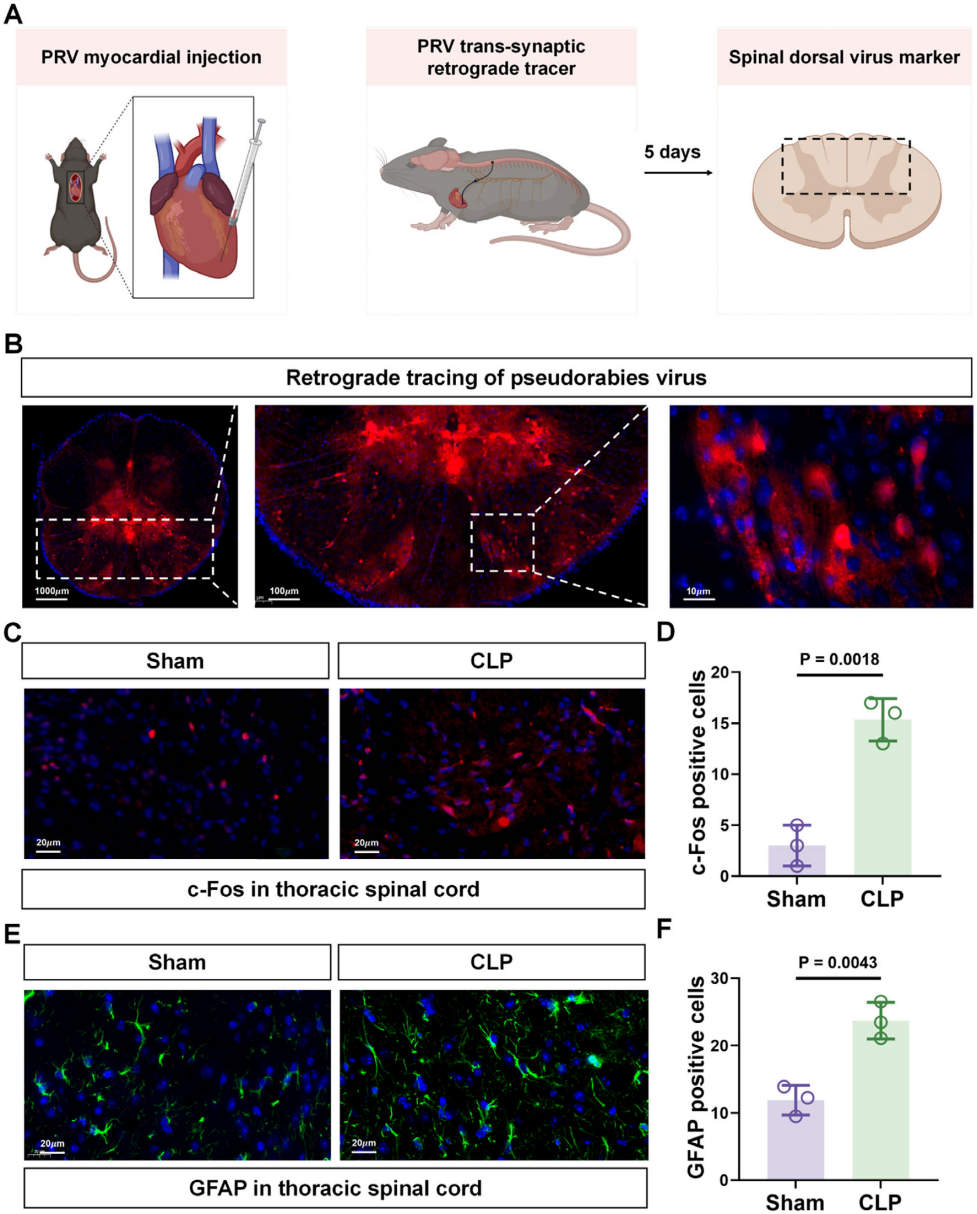
Sepsis activates astrocytes and GABAergic neurons in the thoracic spinal cord. A) Schematic of the experimental procedure. B) Representative epifluorescence image of mCherry expression (red) in thoracic spinal cord sections 4 days after heart injection of PRV to trace synaptic retrograde connectivity. Scale bars: 1000, 100, 10 µm. C,D) Representative epifluorescence images of immunostaining against c‐Fos (red) in thoracic spinal cord sections from Sham and CLP mice. Scale bar: 20 µm (C). Changes in c‐Fos levels in (C) expressed as the percentage of c‐Fos+ cells (n = 3/ sections per group) (D). E,F) Representative epifluorescence images of immunostaining against GFAP (green) in thoracic spinal cord sections from Sham and CLP mice. Scale bar: 20 µm (E). Relative GFAP levels in (E) estimated from fluorescence intensity measurements (n = 3/ sections group) (F). Data are shown as the mean ± SD of three independent experiments and were compared using a two‐tailed, unpaired Student's *t*‐test (D,F).

To investigate the presence of neurons that innervate the heart within the thoracic spinal cord, we performed retrograde tracing by introducing pseudorabies virus into the apex of the ventricular myocardium (**Figure** **2**A). This approach revealed a substantial population of neurons that innervate the heart within the thoracic spinal cord (Figure 2B). Building on this finding, we examined whether these neurons play a role in myocardial injury during sepsis. Immunofluorescence analysis of c‐Fos, a marker of neuronal activation, revealed significantly increased staining in the thoracic spinal cord after CLP compared to sham treatment (Figure 2C,D). Furthermore, akin to prior studies showing that sepsis can activate brain astrocytes,^[^ ^38^ ^]^ we observed a marked increase in astrocytic activation (astrogliosis) in the spinal cord of CLP mice relative to sham controls, as revealed by their increased immunostaining for GFAP (Figure 2E,F). Collectively, these results demonstrate that neurons originating in the dorsal thoracic spinal cord terminate with receptors in the heart. Moreover, we observed that nerve cells and glial cells in the thoracic and dorsal spinal cord were activated following CLP.

### 2.3 TNF‐α‐Mediated GABAergic Neuronal Necroptosis Regulates Septic Cardiomyopathy

**Figure 3:**
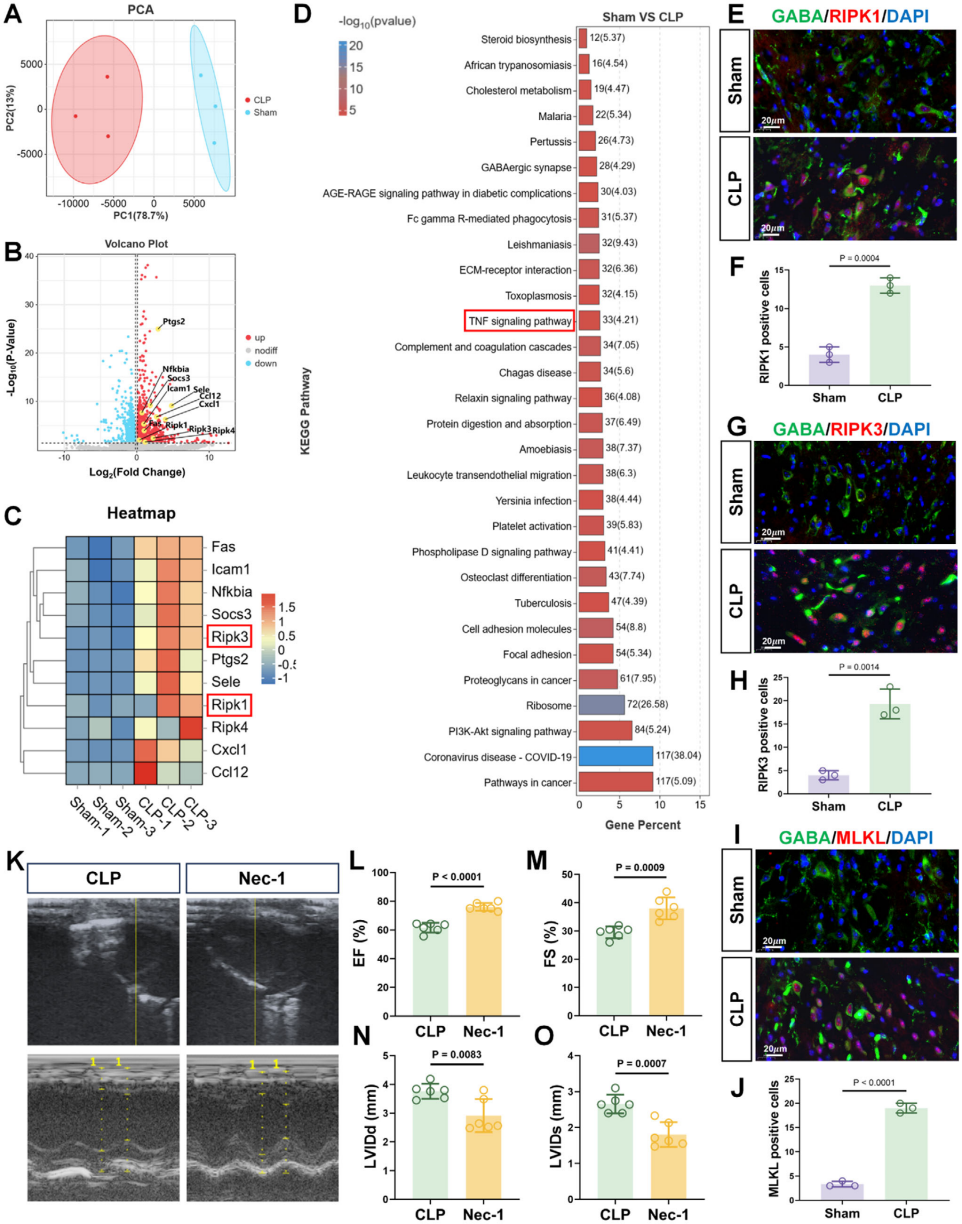
Sepsis induces necrotic apoptosis in thoracic spinal cord GABAergic neurons, with myocardial damage averted by Necrostatin‐1. A–D). Exome sequencing delineating changes in the thoracic spinal cord of Sham and CLP mice. Principal component analysis (PCA) plot showing clustering of replicates, three biological replicates/group; A), Volcano plot showing significantly upregulated and downregulated genes (DEGs; B), top‐ranked KEGG pathway enrichments (C), and heatmap of selected necroptosis‐related genes (D). E–J) Representative epifluorescence images of immunostaining against GABA (green) in combination with RIPK1 (E), RIPK3 (G) or MLKL (I) (red) in thoracic spinal cord sections from Sham and CLP mice. Scale bar: 20 µm. Relative RIPK1 (F), RIPK3 (H), or MLKL (J) levels estimated from fluorescence intensity measurements (n = 3/ sections group). L–O) Representative echocardiograms of CLP operated mice treated without and with Nec‐1 (K), with plots summarizing cardiac EF (L), FS (M), LVIDd (N), and LVIDs (O) (n = 6 mice/group). Data are shown as mean ± SD; two‐tailed Student's *t*‐test.

Transcriptome sequencing comparing sham and CLP‐treated mice was next undertaken to glean clues concerning sepsis‐induced changes in the thoracic spinal cord (**Figure** **3**A). We detected more than 2600 differentially expressed genes (DEGs) with secondary GO analyses showing broad impacts affecting general processes defined by Biological Process, Molecular Function, and GO terms (Figure S1A–C, Supporting Information). Interestingly, the immune system featured among the top signatures enriched through general KEGG and Reactome pathway analyses (Figure S2A,B, Supporting Information) while the top GSEA enrichments also involved numerous immunity‐related gene signatures (Figure S3A–C, Supporting Information). Further interrogation revealed upregulation of key necroptosis regulatory genes, including Receptor‐Interacting Protein Kinase 1 (RIPK1) and Receptor‐Interacting Protein Kinase 3 (RIPK3), in the CLP group compared to sham controls (Figure 3B,C). Moreover, the Tumor Necrosis Factor α (TNF‐α) pathway featured prominently among the top 30 most enriched KEGG pathways (Figure 3D), together inferring increased necroptosis levels. These findings were also reminiscent of reports showing that sepsis and ischemia induce apoptosis in central GABAergic neurons,^[^ ^12^, ^13^, ^14^ ^]^ suggesting in our model that spinal cord GABAergic neurons undergo necroptosis.

Consistent with this, we found that spinal cord GABA levels were reduced in CLP mice compared to controls (Figure S4, Supporting Information) while histological examination of spinal cord tissues after CLP treatment revealed evidence of neuronal death (Figure S5A,B, Supporting Information). Moreover, immunofluorescence detection of the necroptosis activators RIPK1 and RIPK3, along with the terminal effector Mixed Lineage Kinase Domain‐Like protein (MLKL) showed markedly increased co‐staining in spinal cord GABAergic neurons (Figure 3E–J). Thus, sepsis damages thoracic spinal cord GABAergic neurons via necroptosis.

To investigate the connections between necroptosis in GABAergic neurons and sepsis‐induced cardiomyopathy, we treated CLP mice intrathecally with the necroptosis inhibitor Necrostatin‐1 (Nec‐1). Notably, cardiac function was significantly improved in Nec‐1‐treated mice relative to the controls (Figure 3K–O) accompanied by reduced cTnT expression levels in myocardial tissues (Figure S6A–C, Supporting Information). Moreover, Nec‐1 treatment decreased the expression of RIPK1, RIPK3, and MLKL in GABAergic neurons in the spinal cord following Nec‐1 administration (Figure S7A–F, Supporting Information) with corresponding histological evidence showing reduced neuronal apoptosis (Figure S5C, Supporting Information). Importantly, the administration of Nec‐1 via intraperitoneal injections failed to alter the effects of CLP by echocardiographic analysis (Figure S10I–L, Supporting Information), likely ruling out the possibility of drug effects on cardiac function via peripheral circulation and cerebrospinal fluid (CSF) flow.

Collectively these findings propose that necroptosis of GABAergic neurons plays a key role in the pathogenesis of myocardial injury during sepsis.

### 2.4 Sepsis Induces Inflammatory Factor Expression by Astrocytes in the Thoracic Spinal Cord

**Figure 4:**
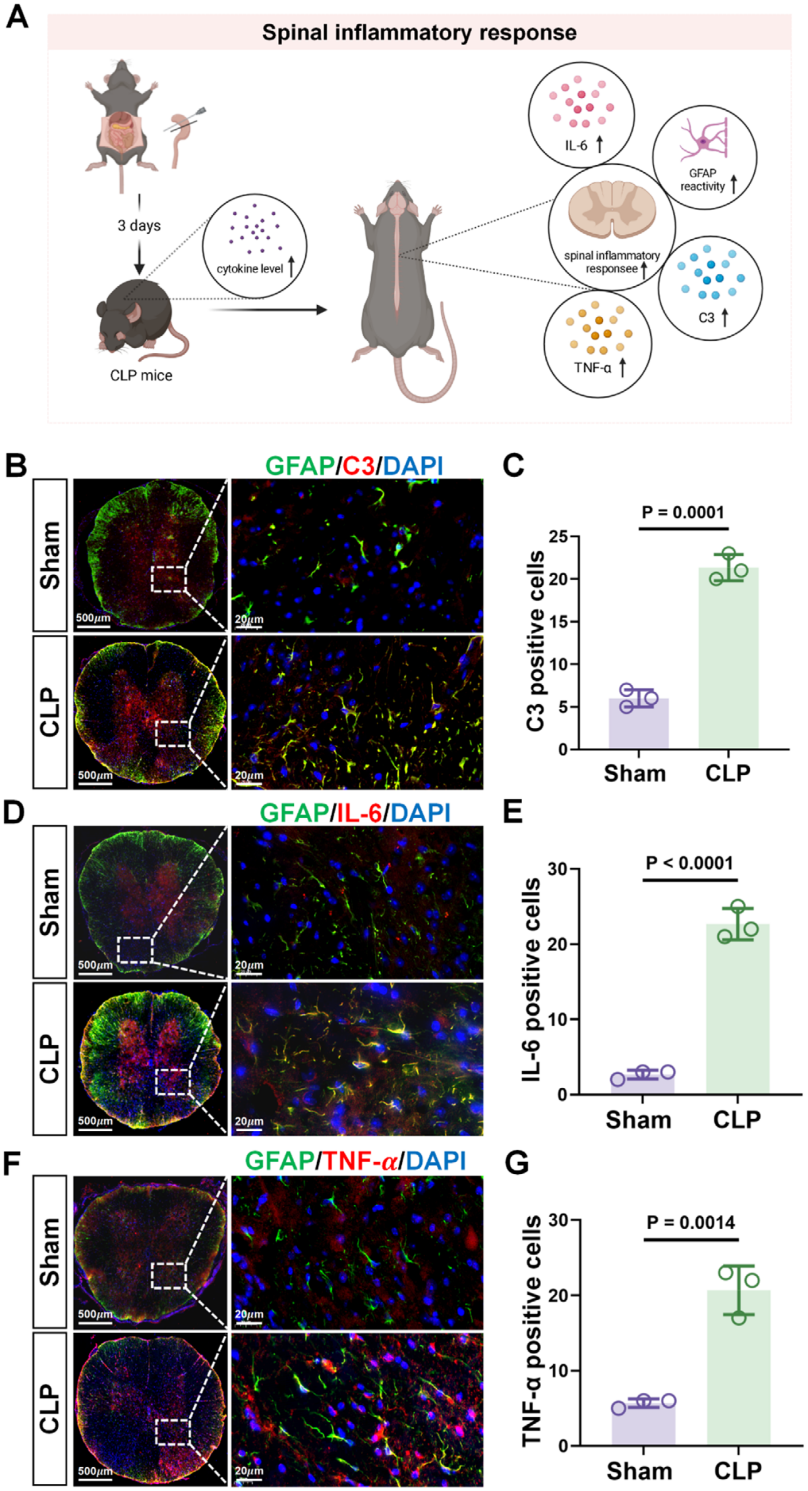
Sepsis induces inflammatory factor production by astrocytes in the thoracic spinal cord. A) Schematic diagram of the experimental procedure. B–G) Representative epifluorescence images of immunostaining against GFAP (green) in combination with complement factor C3 (B), IL‐6 (D), or TNF‐α (F) (red) in thoracic spinal cord sections from Sham and CLP mice. Scale bars: 500 and 20 µm. Relative C3 (C), IL‐6 (E), or TNF‐α (G) levels estimated from fluorescence intensity measurements (n = 3/ sections group). Data are shown as the mean ± SD of three independent experiments and were compared using a two‐tailed, unpaired Student's *t*‐test (C, E, and G).

As the key immune cells in the central nervous system, astrocytes release various inflammatory factors following activation, commonly including complement component 3 (C3), interleukin 6 (IL‐6), and TNF‐α (**Figure** **4**A).^[^ ^47^ ^]^ Transcriptome sequencing analysis indicated changes in numerous immune‐related processes, including significant upregulation of TNF‐α‐related genes following CLP. Confirming the activation of inflammatory responses in the thoracic spinal cord during sepsis, immunofluorescence staining against C3, IL‐6, and TNF‐α revealed marked increases of all three inflammatory markers, with a high degree of overlap observed between astrocytes marked by GFAP (Figure 4B–G). Overall, the above results indicate that spinal cord astrocytes release inflammatory factors during sepsis.

### 2.5 Sepsis Increases Sympathetic Excitability

**Figure 5:**
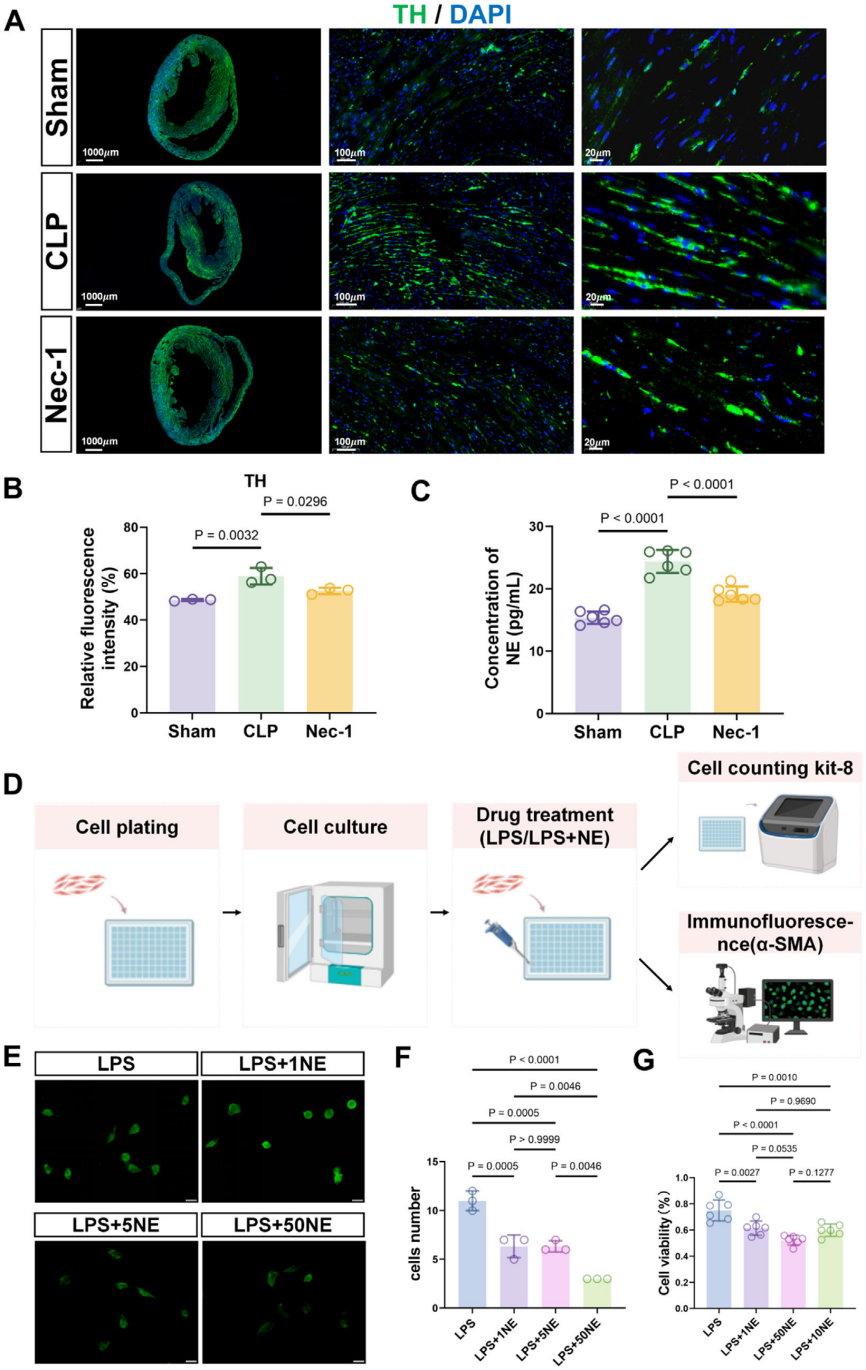
Sepsis enhances cardiac sympathetic activation. A,B) Representative epifluorescence images of immunostaining against tyrosine hydroxylase (TH) (green) and DAPI (blue) staining in heart sections from Sham, CLP, and Nec‐1 mice. Scale bars: 1000, 100, and 20 µm (A). Relative TH levels determined from fluorescence intensity measurements in (a) (n = 3/sections per treatment group) (B). C) Norepinephrine (NE; pg mL^−1^)) plasma concentrations from the treatment groups in (C) (n = 6/mice per treatment group) D) Schematic of the AC16 cell culture model. E–G). AC16 cells were treated with LPS alone or in combination with 1, 5, or 50 pg mL^−1^ NE for 12 h. Representative epifluorescence images of immunostaining against smooth muscle actin (α‐SMA; green) (E), cell number measured by direct counting (F) (n = 3 cultures/group), and cell viability quantified by CCK8 assays (G) (n = 6 wells/group). Data are shown as mean ± SD; two‐tailed Student's *t*‐test and were compared using one‐way ANOVA followed by Tukey's multiple comparisons test (B, C, F, and G).

Sepsis triggers the overactivation of the sympathetic nervous system, leading to the excessive secretion of catecholamines. We therefore reasoned that sepsis would also influence catecholaminergic neurons in myocardial tissues and that such effects could be alleviated through intrathecal administration of Nec‐1. When assessing the presence of tyrosine hydroxylase (TH) expressing sympathetic nerves using immunofluorescence, we observed significant increases in TH‐positive signals in CLP mice compared to the sham group. Moreover, administration of Nec‐1 noticeably reduced the abundance of sympathetic nerve endings in CLP mice (**Figure** **5**A,B). Furthermore, increased plasma norepinephrine levels were evident in the CLP‐treated mice, which were effectively reduced after Nec‐1 treatment (Figure 5C). Together, these results suggest that excessive sympathetic nervous system excitation caused by sepsis is effectively suppressed by Nec‐1.

As a corollary to these findings, we modeled the effects of sepsis and excessive sympathetic activation in vitro. AC16 cardiomyocytes were treated with endotoxin (LPS) alone or in combination with varying concentrations of norepinephrine (NE) (Figure 5D). Compared to treatment with LPS alone, the addition of NE resulted in a reduction in cell numbers, as evidenced by smooth muscle actin (SMA) staining (Figure 5E). These findings were corroborated by cell counting assays, which showed a progressive decline in total cell numbers with increasing NE concentrations (Figure 5F). Furthermore, CCK‐8 assays demonstrated dose‐dependent decreases in cell viability with NE treatment (Figure 5G). Corroborating these findings, LDH release assays showed dose‐dependent increases in LDH release with increasing NE concentrations (Figure S11, Supporting Information). Collectively, these results indicate that excessive norepinephrine exacerbates the cytotoxic effects of LPS on cardiomyocytes.

### 2.6 Dexmedetomidine Alleviates Sepsis‐Induced Myocardial Injury by Inhibiting Astrocyte Activation and GABAergic Neuronal Necroptosis

**Figure 6:**
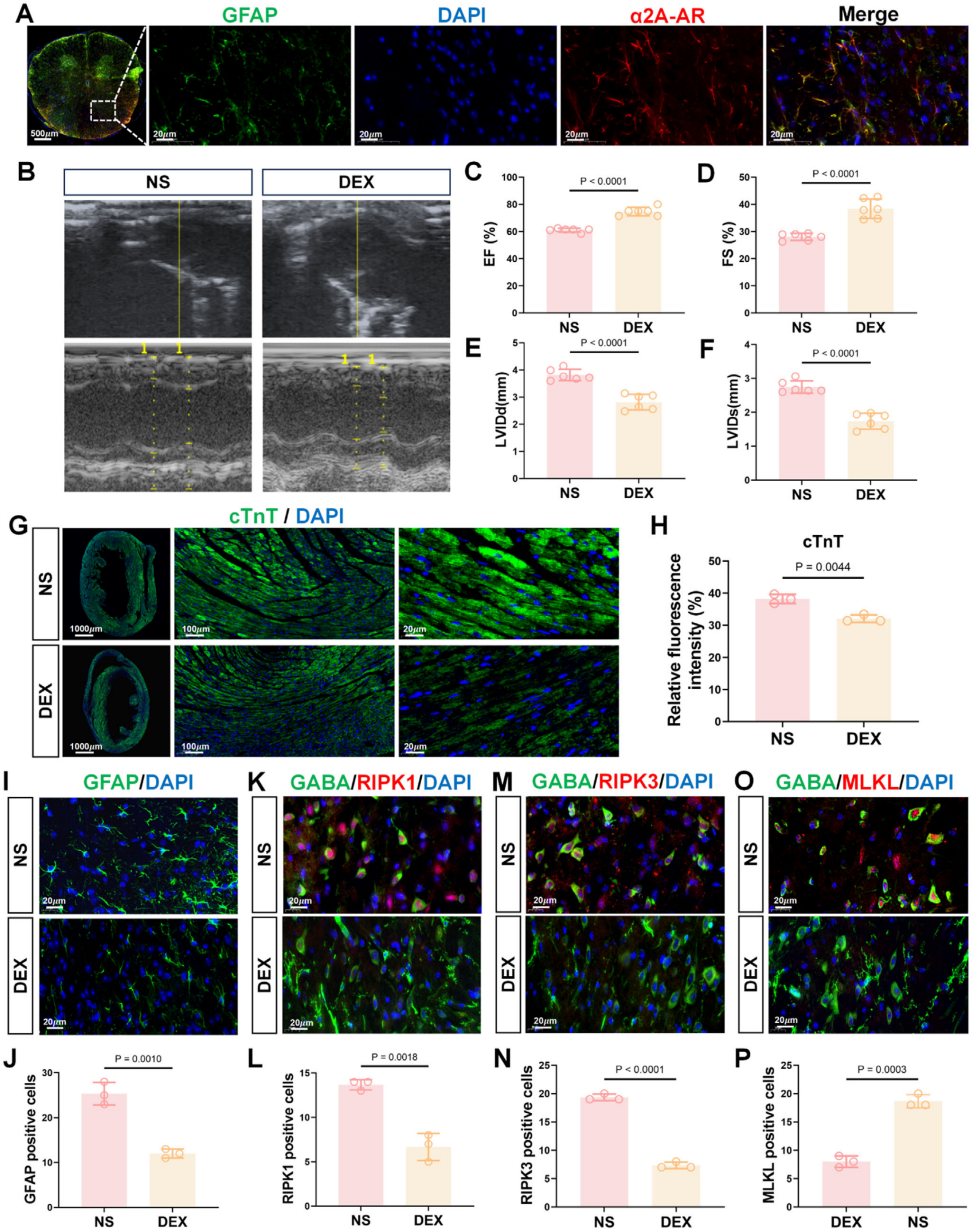
Dexmedetomidine alleviates sepsis‐mediated myocardial damage by reducing astrocytic activation and neuronal necroptosis. A) Representative image showing expression of the α2A in the thoracic spinal cord. Scale bars: 500 µm, 20 µm. B–F) Representative echocardiograms of CLP operated mice treated with normal saline (NS) or dexmedetomidine (DEX) (B), with plots summarizing cardiac EF (C), FS (D), LVIDd (E), and LVIDs (F) measurements (n = 6 mice per group). G,H) Representative epifluorescence images of immunostaining against cTnT (green) in myocardial tissue sections from mice in the NS and DEX treatment groups. Scale bars: 1000, 100 and 20 µm (G). Relative cTnT levels in (G) estimated from fluorescence intensity measurements (n = 3/sections per group) (H). I,J) Representative epifluorescence images of immunostaining against GFAP (green) along with DAPI staining (blue) in thoracic spinal cord sections from NS and DEX mice. Scale bars: 20 µm (I). Changes in GFAP in (I) expressed as the percentage of GFP+ cells (n = 3/sections per group) (J). K, M, and O) Representative epifluorescence images of immunostaining against GABA (green) in combination with RIPK1 (K), RIPK3 (M), or MLKL (O) (red) in thoracic spinal cord sections from NS and DEX mice. Scale bars: 20 µm. Relative RIPK1 (L), RIPK3 (N), or MLKL P) levels estimated from fluorescence intensity measurements (n = 3/ sections per group). Data are shown as the mean ± SD of three multiple independent experiments and were compared using two‐tailed, unpaired Student's t‐test (C–F, H, and J–P).

Notably, we identified prominent α2A‐AR expression on astrocytes in the thoracic spinal cord of mice (**Figure** **6**A) and hypothesized that dexmedetomidine may have protective effects against septic cardiomyopathy. In support of this idea, intrathecal administration of dexmedetomidine significantly improved cardiac function following CLP, as indicated by increased EF and FS along with reduced LVIDd and LVIDs (Figure 6B–F). Control experiments administering dexmedetomidine via intraperitoneal injections showed no significant alterations in echocardiographic parameters in the CLP only group (Figure S10I–L, Supporting Information), supporting the notion of the localized effects of intrathecal administration.

Accompanying histological examination showed dexmedetomidine decreased inflammatory cell infiltration in the myocardium (Figure S10A,B, Supporting Information) in conjunction with reduced apoptosis in spinal cord neurons (Figure S5D,E, Supporting Information) along with reducing heightened cardiac troponin levels (Figure 6G,H). Moreover, dexmedetomidine significantly inhibited the activation of thoracic spinal cord astrocytes (Figure 6I,J) and lowered their levels of inflammatory markers (Figure S9A–F, Supporting Information) with accompanying reductions in the expression of necroptosis‐related markers in GABAergic neurons (Figure 6K–P). These findings suggest that dexmedetomidine mitigates myocardial injury in sepsis by suppressing astrocyte activation and reducing neuronal necroptosis in the spinal cord.

### 2.7 Astrocyte α2A Receptors Mediate the Myocardial Protective Effect of Dexmedetomidine

**Figure 7:**
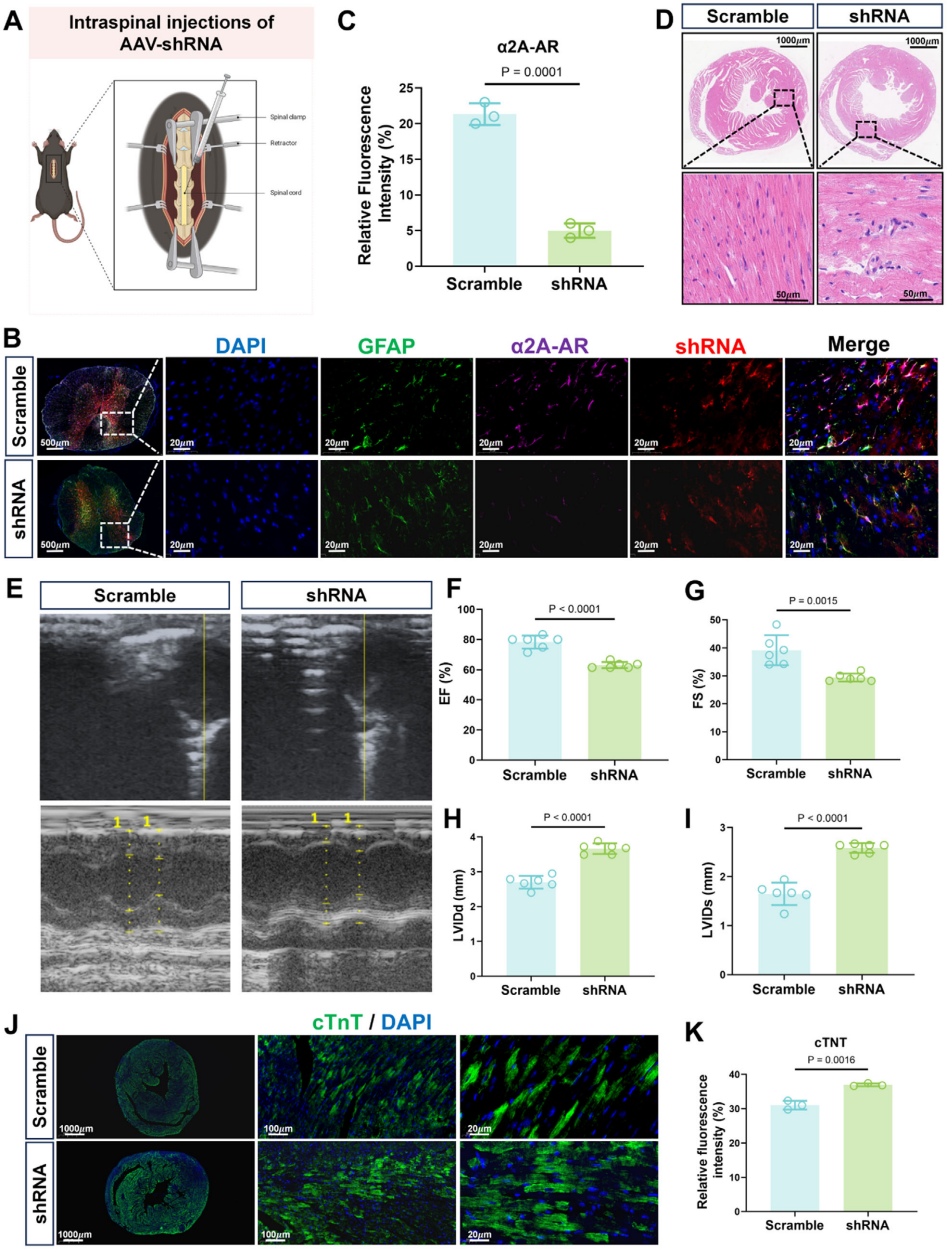
Adenoviral‐mediated knockdown of α2A‐AR expression reverses the myocardial protective effects of dexmedetomidine. A) Schematic illustrating spinal cord injection procedure for AAV‐shRNAs. B) Representative histological (HE) micrographs of heart sections from CLP mice pretreated with control (Scramble) or α2A‐AR targeting shRNAs (shRNA) in combination with dexmedetomidine. scale bars: 1000 and 20 µm. C,D) Representative epifluorescence images of DAPI (blue), GFAP (green), α2A‐AR (violet), and AAV (mCherry; red) staining in thoracic spinal cord sections from Scramble and shRNA group mice. Scale bars: 20 µm (C). Relative α2A‐AR protein levels estimated from fluorescence intensity measurements in (c) (n = 3/sections per treatment group) (D). E–I) Representative echocardiograms of Scramble and shRNA group mice (E), with plots summarizing cardiac EF (F), FS (G), LVIDd (H), and LVIDs (I) measurements (n = 6 mice/group). J,K) Representative epifluorescence images of cTnT (green) and DAPI (blue) staining in heart sections from Scramble and shRNA group mice. Scale bars: 20 µm (J). Relative cTnT protein levels estimated from fluorescence intensity measurements in (J) (n = 3/sections per treatment group) (K). Data are shown as mean ± SD; two‐tailed Student's *t*‐test (C, F–I, and K).

**Figure 8:**
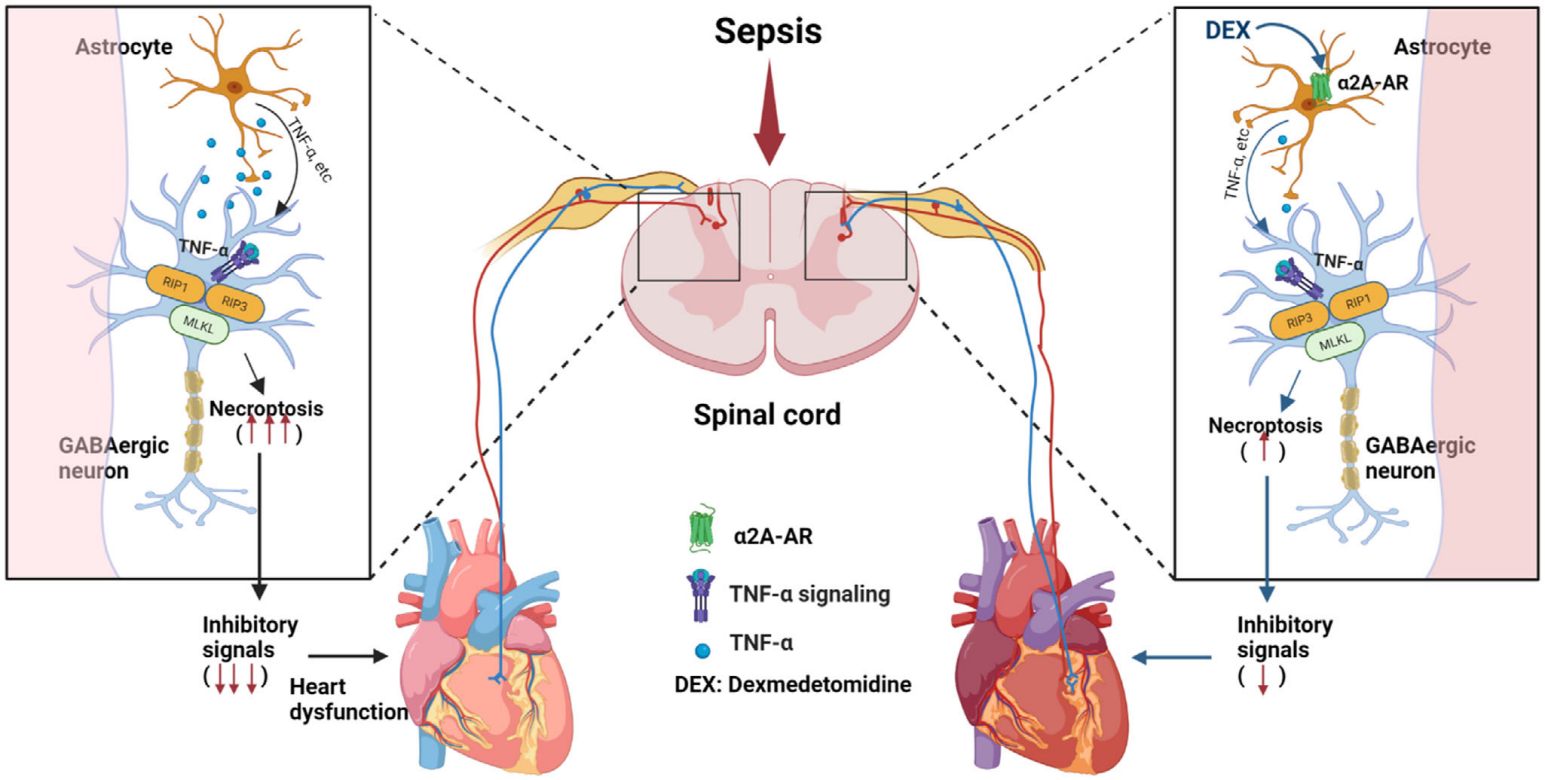
Proposed model. Sepsis‐induced myocardial injury and functional decline result from GABAergic neuronal necroptosis mediated through spinal dorsal horn astrocytic activation and inflammatory factor release (left). Dexmedetomidine can reduce or prevent sepsis‐related phenotypes via α2A‐AR stimulation to prevent the activation of spinal dorsal horn astrocytes.

To verify that the cardioprotective effects of dexmedetomidine involved bona fide effects on α2A‐AR expressed by astrocytes, we administered shRNA‐bearing adenoviruses to target α2A‐AR expression via an intrathecal route (**Figure** **7**A). Confirming the effectiveness of this approach, we observed significant reductions in α2A‐AR expression by thoracic spinal cord astrocytes (Figure 7C,D). Importantly, compared to the scramble shRNA control mice, there were marked increases in myocardial inflammatory cell infiltration after α2A‐AR knockdown in CLP mice treated with dexmedetomidine (Figure 7B). Consistently, echocardiographic analysis demonstrated comparative decreases in EF and FS, along with increased LVIDd and LVIDs in the α2A‐AR shRNA‐treated mice (Figure 7E–I), along with increased cardiac troponin levels (Figure 7J–K) and sympathetic nerve terminal densities (Figure S8A,B, Supporting Information). Moreover, like knockdown of α2A‐AR, alternative pharmacological intervention using the α2A‐adrenoceptor inhibitor BRL‐44408 maleate (BRL‐8) in CLP mice served to antagonize the benefits of dexmedetomidine, causing a relative decline in cardiac function (Figure S10E–H, Supporting Information) associated with increased neuronal loss in the spinal cord (Figure S5B,E,F, Supporting Information), increased TH‐positive signals (Figure S8A,B, Supporting Information), cardiac troponin staining (Figure S6B,C, Supporting Information) and myocardial inflammatory cell infiltration (Figure S10B,D, Supporting Information). Together, these findings suggest that dexmedetomidine exerts its cardioprotective effects by inhibiting astrocyte activation via the α2A‐AR in the thoracic spinal cord. Our working model is illustrated in **Figure** **8**.

## 3 Discussion

Sepsis causes life‐threatening organ dysfunction through a dysregulated host response to infection and is often accompanied by cardiomyopathy and high mortality.^[^ ^1^ ^]^ Previous work in a rat model of myocardial ischemia‐reperfusion showed that inhibiting astrocyte activation in the spinal cord alleviates myocardial ischemic injury.^[^ ^48^ ^]^ Furthermore, hippocampal astrocytes are activated during sepsis‐associated encephalopathy, resulting in cognitive dysfunction and impaired learning and memory.^[^ ^38^, ^49^ ^]^ However, whether the activation of spinal astrocytes plays any significant role in septic cardiomyopathy remains an open question. Here we demonstrate that spinal dorsal horn astrocytes are activated during sepsis in mice, as indicated by GFAP upregulation and marked production of inflammatory factors such as TNF‐α. Our study predominantly identified manifestations of myocardial dysfunction, accompanied by inflammatory cell infiltration within the myocardium and elevated troponin levels. While previous research has extensively characterized cardiac dysfunction in sepsis, structural myocardial necrosis has not been considered as a characteristic feature in either clinical cases of sepsis or CLP models.^[^ ^17^, ^50^ ^]^ We connect this activation with downstream events leading to myocardial damage, providing proof‐of‐principle strategies that could limit or reverse sepsis‐related heart damage.

Clinical and preclinical studies suggest that α2‐adrenergic receptor agonists such as dexmedetomidine can help to stabilize hemodynamics and provide cardiovascular protection in septic patients. During the early stages of sepsis, central sympathetic nervous system function modulated by α2 agonists may be beneficial, as it suppresses cardiac sympathetic nerve activity, thereby reducing heart rate, contractility, cardiac output, myocardial oxygen consumption, and lactate release.^[^ ^39^ ^]^ Previous research demonstrated that dexmedetomidine provides neurocognitive protection from sepsis through effects on α2A‐AR in the central nervous system.^[^ ^38^ ^]^ Here we found that, similar to hippocampal astrocytes,^[^ ^51^ ^]^ dorsal horn astrocytes express α2A receptors. Importantly, intrathecal dexmedetomidine inhibited spinal astrocyte activation, GFAP upregulation in CLP mice, while also limiting sympathetic activation. This led to significant improvements in cardiac contractile dysfunction, indicative that dexmedetomidine alleviates sepsis‐induced cardiomyopathy during sepsis. Importantly, when α2A‐AR expression or activity in spinal astrocytes was selectively downregulated or blocked, sympathetic excitability remained elevated, and the cardioprotective actions of intrathecal dexmedetomidine were lost. Thus, the benefits of dexmedetomidine result from direct activation of α2A‐AR on spinal astrocytes. Considering these findings, this study suggests a model of spinal cord regulation of neuroinflammation and peripheral cardiac dysfunction in sepsis that incorporates previously demonstrated concepts of astrocyte regulation of inflammation and the anti‐inflammatory effects of dexmedetomidine. Moreover, the data support the existence of a novel and previously unrecognized heart–spinal cord–immune axis in sepsis that can exert protective effects on remote organ systems.

Prior studies identify that astrocytes regulate necroptosis in spinal dorsal horn neurons, with astrocytes playing a role in excitotoxicity, a mechanism in Alzheimer's disease (AD), amyotrophic lateral sclerosis (ALS), and Huntington's disease (HD), where GABAergic neurotransmission is reduced.^[^ ^24^ ^]^ Moreover, GABAergic neuronal apoptosis leads to reduced central inhibitory function and an imbalance in excitatory‐inhibitory signaling, which plays a critical role in cardiovascular regulation. Astrocytes regulate GABAergic neuronal activity responses,^[^ ^52^ ^]^ with our previous work demonstrating that upregulating GABAARδ in the thoracic spinal cord significantly reduced myocardial infarction area, arrhythmia, and cardiomyocyte apoptosis after myocardial ischemia.^[^ ^14^ ^]^ Here we show that CLP‐induced sepsis increases necroptosis in dorsal horn spinal cord GABAergic neurons, likely mediated by the release of TNF‐α from activated astrocytes, well known to trigger necroptosis through death receptor engagement.^[^ ^53^ ^]^

This notion is supported by the enrichment of TNF‐α signaling pathway by transcriptome sequencing, with confirmation of protein level expression changes in key necroptosis‐related genes. Within spinal GABAergic neurons, we observed increases in RIPK1 and RIPK3, which cooperate to establish the necrosome, along with the downstream effector MLKL, responsible for disrupting membrane integrity and causing cell rupture.^[^ ^54^ ^]^ Furthermore, the essential role of necroptosis by intrathecal administration of Nec‐1 alleviated the necroptotic changes observed in GABAergic neurons. Moreover, akin to the effects of dexmedetomidine, we found that Nec‐1 treatment also prevented sympathetic overactivity and cardiac dysfunction. Interestingly, dexmedetomidine has previously been shown to reverse neurodegenerative changes and neuronal apoptosis, likely through the inhibition of apoptotic pathways. For example, in rat models, dexmedetomidine demonstrated neuroprotective effects in sepsis by mitigating inflammatory responses.^[^ ^55^, ^56^ ^]^ Notably, we found that intrathecal administration of dexmedetomidine similarly protected against GABAergic neuronal damage, thus supporting a hierarchy where astrocyte activation precedes neuronal necroptosis during sepsis.

Lastly, our study confirmed that the deleterious effects of sepsis fundamentally rely upon the neural network connections between the central spinal cord and the heart. We formally demonstrated these connections using retrograde labelling where viruses introduced into the myocardium were observed to label central spinal neurons in the thoracic segment, including GABAergic neurons. Treatments with either Nec‐1 or dexmedetomidine were found to effectively inhibit sympathetic overactivity and cardiac dysfunction. Such findings appear to be consistent with prior work showing that activation of GABAergic signaling following spinal cord stimulation reduces sympathetic excitation and ventricular arrhythmias induced by myocardial ischemia‐reperfusion.^[^ ^13^ ^]^

However, our study has further limitations that should be acknowledged, which may influence the interpretation of the experiments as well as limit their clinical applicability. First, our study used a CLP‐induced murine model of sepsis, which is not an exact representation of human sepsis nor sepsis‐induced cardiomyopathy. Thus, further assessment of the safety, efficacy, and long‐term benefits of dexmedetomidine and Nec‐1 in reducing other causes and types of cardiomyopathy is implicitly required before considering the extrapolation of these data into the clinical setting. Furthermore, it should be acknowledged that we omitted antibiotic use in the CLP mouse model, which is often administered to better mimic clinical sepsis. Nevertheless, while the exclusion of antibiotics allowed us to focus on early inflammatory responses against severe, untreated infection, this distinction may be important in comparison to other reported findings. Another related point to consider involves the choice of young male mice in the model, which cannot adequately capture the complexity of the clinical disease involving pathophysiological heterogeneity associated with age and sex. From the biological perspective, our findings invoke a major role for necroptosis, although we cannot presently exclude if other forms of programmed cell death are also involved.

Moreover, while our experiments highlight the importance of factors including C3, IL‐6, and TNF‐α, it is certain that additional players, both the brain and spinal cord, are involved. Consequently, additional work is needed to provide further insights, particularly to understand the integration of signals leading to disease progression. We also did not examine the impact of higher central nervous system functions on septic cardiomyopathy or the effects of these interventions on other organ systems besides the heart. Finally, while the relevance of our findings to human disease requires further evaluation, our results lay a strong foundation for exploring the neuroimmune axis as a potential therapeutic target for treating septic cardiomyopathy.

In conclusion, our findings indicate that in a CLP‐induced murine model of sepsis, spinal astrocyte activation and neuroinflammation induce necroptosis of spinal GABAergic neurons, trigger excessive excitation of the sympathetic nervous system, and ultimately lead to myocardial injury and functional decline. Activating α2A‐AR may prevent astrocyte activation in the spinal dorsal horn, inhibit necroptosis of spinal GABAergic neurons, and reduce the incidence of sepsis‐induced myocardial injury and functional decline (Figure 8). Together, these observations suggest a previously underexplored neuroimmune pathway that may be involved in the pathophysiology of septic cardiomyopathy. This study broadens mechanistic insights into central regulation of cardiac dysfunction during sepsis, potentially informing future research directions.

## 4 Experimental section

Mice were randomly assigned to one of eight treatment groups, consisting of six animals each. Experiments involved sham operated mice (Sham), CLP operated mice (CLP); CLP mice intrathecally administered normal saline (NS); CLP mice intrathecally administered Necrostatin‐1 (Nec‐1, Cat. No.: HY‐15760; MCE); CLP mice intrathecally administered Dexmedetomidine (DEX, Yangtze River Pharmaceutical, Taizhou, China); mice intrathecally administered α2A‐AR‐targeting adenoviruses 21 days prior to CLP and intrathecal administration of DEX (shRNA); mice receiving intrathecal administration of control shRNA‐bearing adenoviruses 21 days prior to CLP and intrathecal administration of DEX (Scramble); CLP mice intrathecally administered Dexmedetomidine and BRL‐44408 maleate (BRL‐8, Cat. No.: HY‐12716A; MCE). Mice were given three times of each pharmacological agent (150 ug kg^−1^) or normal saline via intrathecal catheter delivery, immediately after surgery, and again 24 and 48 h post‐operatively.

### Animal Model of Cecal Ligation and Puncture

Male C57BL/6 mice (8 weeks old) were purchased from the GemPharmatech Co., Ltd. and kept in quarantine for one week to allow for adaptation to the environmentally controlled barrier conditions (temperature: 20–25 °C; Humidity: 50 ± 5%). Chow was provided ad libitum. Welfare and experimental procedures were performed in accordance with the Ethical Regulations on the Care and Use of Laboratory Animals of Anhui Medical University under approval from the school committee for animal experiments (no. 20240192). Mice received CLP surgery as described to induce mid‐grade sepsis.^[^ ^51^, ^57^, ^58^ ^]^ Briefly, after anesthesia with 2% isoflurane, mice were immobilized on an aseptic operating table fitted with a heating pad and the abdominal area depilated and disinfected. Thereafter, the cecum was exposed and isolated through a 1 cm abdominal mid‐line incision before ligation and puncture with a 22‐gauge needle at the distal a‐half of the cecum. Pressure was applied to exude fecal contents through the puncture site before repositioning the cecum in the abdominal cavity and closing the wound with a 4–0 silk suture. The surgical site was infiltrated with 0.25% bupivacaine. Another skin disinfection procedure was performed, and 50 mL kg^−1^ 0.9% saline was injected subcutaneously after surgery. Sham groups underwent the same surgical procedure except caecal ligation and puncture.

### Echocardiography

Echocardiography assessments were performed on mice under isoflurane anesthesia using a VINNO 6 vet instrument with cardiac function parameters recorded, including ejection fraction (EF), fractional shortening (FS), Left Ventricular Internal Diameter at End‐diastole (LVIDd), and Left Ventricular Internal Diameter at End‐systole (LVIDs).

### Cell Culture

The human cardiomyocyte‐like cell line AC16 obtained from Chinese Academy of Sciences Shanghai Institute of Materia Medica (Shanghai) were cultured in Dulbecco's Modified eagle medium (DMEM) supplemented with 10% foetal bovine serum, 100 µg mL^−1^ streptomycin and 100 U mL^−1^ penicillin and maintained at 37 °C in a humidified atmosphere of 95% O_2_, 5% CO_2_.

### Cell Viability Assay

Viability assessments were performed using the Cell Counting Kit 8 assay (CCK‐8; Beyotime, China). AC16 cells were seeded into 96 well plates at 3000 cells well^−1^ in 100 µL complete culture medium. Norepinephrine (NE) and LPS dissolved in DMSO and DEPC‐treated water, respectively, were diluted with culture medium and added to the cells for 12 h before the addition of 10 µl well^−1^ of CCK‐8 solution. After 1 h further incubation, optical densities at 450 nm were measured using a microplate reader (PerkinElmer, Turku, Finland) with data expressed as normalized cell viability measurements.

### Lactate Dehydrogenase Cytotoxicity Assay

Cytotoxicity assessments were performed using the LDH Cytotoxicity Assay Kit (LDH; Beyotime, China) according to the manufacturers’ instructions. Briefly, AC16 cells were seeded into 96 well plates at 3000 cells/well in 100 µL complete culture medium. Norepinephrine (NE) and LPS dissolved in DMSO and DEPC‐treated water, respectively, were diluted with culture medium and added to the cells for 12 h. LDH release reagent (10% of the original culture medium volume) was added 1 h prior to collecting culture supernatants and performing detection in a separate 96‐well plate.

### Immunofluorescence Staining of Tissues and Cells

Immediately after sacrifice, microsurgical forceps were used to remove bone and expose the thoracic spinal cord before removal. Tissues were immediately fixed in 4% formaldehyde solution at 4 °C overnight before sequential dehydration of tissues in 15% and 30% sucrose‐PBS solutions. The tissues were then embedded in optimal cutting temperature compound (cat. no. 4583; Sakura, Japan) before cutting 10 µm sections using a cryostat microtome (SLEE, Germany). The slides were then fixed in 4% formaldehyde for 10 min, and blocked with 3% BSA+ 0.3% Triton‐100 PBS for 1 h at 37 °C before overnight incubation at 4 °C with either chicken polyclonal anti‐GFAP antibody (1:500 dilution, Cat. No.: ab4674; Abcam), rabbit polyclonal anti‐C3 antibody (1:200 dilution, Cat. No.: ab11887; Abcam), rabbit monoclonal anti‐IL‐6 antibody (1:200 dilution, Cat. No.: ab290735; Abcam), mouse monoclonal anti‐TNF antibody (1:200 dilution, Cat. No.: ab1793; Abcam), c‐Fos antibody (E‐8) (1:200 dilution, Cat. No.: sc‐166940; Santa Cruz), mouse monoclonal anti‐GABA antibody (1:200 dilution, Cat. No.: ab86186; Abcam), rabbit polyclonal antibody to Phospho‐RIPK1 (1:200 dilution, Cat. No.: AF2398; Affinity), rabbit polyclonal antibody to Phospho‐MLKL (1:200 dilution, Cat. No.: AF2398; Affinity), rabbit polyclonal antibody to Phospho‐RIPK3 (1:200 dilution, Cat. No.: AF2398; Affinity), rabbit polyclonal antibody to TH (1:200 dilution, Cat. No.: 25859‐1‐AP; Proteintech), rabbit polyclonal antibody to Cardiac Troponin T (1:200 dilution, Cat. No.: 15513‐1‐AP; Proteintech). After washing three times with PBS for 5 min each, sections were incubated with species matched fluorophore‐conjugated secondary antibodies (Alexa Fluor 568, Alexa Fluor 488, or Alexa Fluor 647, Invitrogen, all diluted 1:500) at RT for 2 h, followed by washing three times with PBS and mounting using Antifade Mounting Medium with DAPI (Cat. No.: P0131‐25 mL; Beyotime Biotechnology). Multi‐channel epifluorescence images were acquired using a 3DHISTECH whole slide scanner and quantified using the Measure function in ImageJ software. Alternatively, AC16 cells cultured on glass slides and subjected to the indicated treatments were fixed in 4% formaldehyde for 30 min, before blocking non‐specific binding with 10% normal goat serum. Thereafter, primary antibodies against alpha SMA diluted 1:300 (mouse monoclonal, Cat. No.: 67735‐1‐lg; Proteintech) were incubated overnight at 4 °C before detection with Alexa Fluor 568 secondary antibodies (Invitrogen, USA) for 2 h at RT. Follow the methods of random sampling and double‐blind procedure when collecting statistical images. Cell nuclei were counterstained with DAPI and digital micrographs acquired using an epifluorescence microscope.

### Pseudorabies Virus (PRV) Trans‐Synaptic Retrograde Tracer Technique

Mice were given an intraperitoneal injection of pentobarbital sodium (40 mg kg^−1^). Mechanical ventilation was established after fixing the upper teeth and limbs and introducing an indwelling needle into the airway connected to a ventilator (respiratory ratio 1:1, tidal volume 1–1.5 mL, respiratory frequency 120–130 min^−1^). Afterward, the left fourth and fifth intercostal spaces were opened before stripping the pericardium to expose the apex of the heart. A micro‐syringe and injection needle were fixed in place in the left region of the ventricular myocardium, and PRV (PRV‐CAG‐EGFP, BrainVTA, Wuhan, China) was injected into three sites for 10 min each (6 × 109 pfu mL^−1^, 0.5 µl site^−1^). The mice were sacrificed 5–7 days after PRV injection, and spinal tissues collected, fixed, and sectioned as described above. PRV tracing was assessed by EGFP fluorescence.

### Spinal Cord Sheath Built‐In Tube

Following anesthesia with sodium pentobarbital, mice were positioned prone before making a 1 cm longitudinal skin incision along the midline of the neck (at the level of the occipital protuberance) and the muscles pushed aside to expose the atlanto‐occipital membrane. Under microscopic visualization, a 32G needle was used to puncture the atlanto‐occipital membrane before introducing a polyethylene‐10 catheter into the cavity in the tail direction until reaching the thoracic spinal cord. The distal end of the catheter was then sutured to the muscle and buried beneath the skin before introducing drugs and viruses into the spinal canal through the catheter.

### Hematoxylin‐Eosin Staining

Spinal and cardiac tissues were fixed in 4% formaldehyde solution after removal and subsequent embedding in paraffin. Five µm sections were cut, dewaxed in xylene, and rehydrated in graded alcohol solutions before conducting hematoxylin staining for 5 min, differentiated in 1% hydrochloric acid alcohol for 20 s before Eosin staining for 2 min. The sections were mounted following standard dehydration and clarification procedures, and whole slide images of the tissues were acquired using a 3DHISTECH scanner.

### Enzyme‐Linked Immunosorbent Assays

Norepinephrine concentrations in mouse serum and the GABA concentration in spinal cord CSF were measured using the Mouse Noradrenaline ELISA Kit (CSB E07870m; Cusabio) and the pan‐species ELISA Kit for Gamma‐Aminobutyric Acid (CEA900Ge; Cloud‐Clone Corp), respectively, according to the manufacturers’ instructions.

### Intraspinal Injection of AAV‐shRNA

Injections were performed at the T2‐T6 level of the thoracic spinal cord as described previously.^[^ ^48^ ^]^ Each animal received a single microinjection of either 5ul of shRNA‐bearing adenoviruses targeting murine α2A‐AR (rAAV‐GFaABC1D‐mCherry‐5′miR‐30a‐shRNA (Adra2a)‐3′miR‐30a‐WPRE) or a scrambled control shRNA (rAAV‐GFaABC1D‐mCherry‐5′miR‐30a‐shRNA (scramble)‐3′miR‐30a‐WPRE) (BrainVTA, Wuhan, China). The final viral titres of rAAV‐GFaABC1D‐mCherry‐5′miR‐30a‐shRNA (Adra2a)‐3′miR‐30a‐WPREs were 5.69E+12 vg mL^−1^ and 5.52E+12 vg mL^−1^. Mice were subjected to cecal ligation and puncture 21 days post‐injection, a period predetermined as sufficient for shRNA interference.

### Statistical Analysis

Data were analyzed using GraphPad Prism v9.5 (GraphPad Software, San Diego, CA, USA). Unpaired Student's *t*‐test was used for comparisons between two independent groups. Data were subjected to one‐way ANOVA followed by Tukey's multiple comparisons test if they passed the Shapiro–Wilk test. Data were presented in the text as the mean and standard deviation (SD) in the form “mean ± SD”. A *p* value<0.05 was considered statistically significant.

## Conflict of Interest

The authors declare that no conflicts of interest exist.

## Author Contributions

R.H., B.W., and L.H. contributed equally to this work. Y.L., X.L., B.M., B.W., and R.H. conceived the study. R.H., B.W., and L.H. performed the majority of the experiments and wrote the first draft of the article. R.H., L.H., and J.F. performed the animal model. M.L. and W.G. contributed to the bioinformatics experiments and analysis. B.W., J.F., and B.M. contributed to data analysis. Y.L., X.L., and E.G. revised the article.

## Supporting information



Supporting Information

## Data Availability

The data that support the findings of this study are available from the corresponding author upon reasonable request.
