# Supplementary material for: Activation of Spinal Astrocyte α2A Adrenoceptors Protects Against Sepsis‐Induced Heart Injury Through Inhibition of GABAergic Neuronal Necroptosis
Source: Adv Sci (Weinh). 2025 Jul 21;12(39):e04406. doi: 10.1002/advs.202504406 (PMC12533331; doi:10.1002/advs.202504406)
Supplement: Supplementary file 1 — Supporting Information [file ADVS-12-e04406-s001.docx]

**Supplementary Materials**

Figures. S1 to S11

Table S1

**Activation of Spinal Astrocyte α2A Adrenoceptors Protects against Sepsis-Induced Heart Injury through Inhibition of GABAergic Neuronal Necroptosis**

Ruilin He^1#^, Bin Wu^1#^, Liuhu Han^1#^, Mingde Li^1^, Wenli Guo^1^, Jiajing Fu^1^, Bin Mei^1^, Eric R. Gross^2^, Xuesheng Liu^1,*^, Yao Lu^1,*^

1 Department of Anesthesiology, The First Affiliated Hospital of Anhui Medical University, Key Laboratory of Anesthesia and Perioperative Medicine of Anhui Higher Education Institutes, Anhui Medical University, Hefei 230022, China.

2 Department of Anesthesiology, Perioperative and Pain Medicine, School of Medicine, Stanford University, 94305, CA

# These authors contributed equally to this work.

* Corresponding authors. E-mail: Yao Lu, luyao@ahmu.edu.cn; Xuesheng Liu, liuxuesheng@ahmu.edu.cn.

*.*

**This file includes:**

Figures. S1 to S11

Tables S1

**Supplemental Figure S1-S11**

**
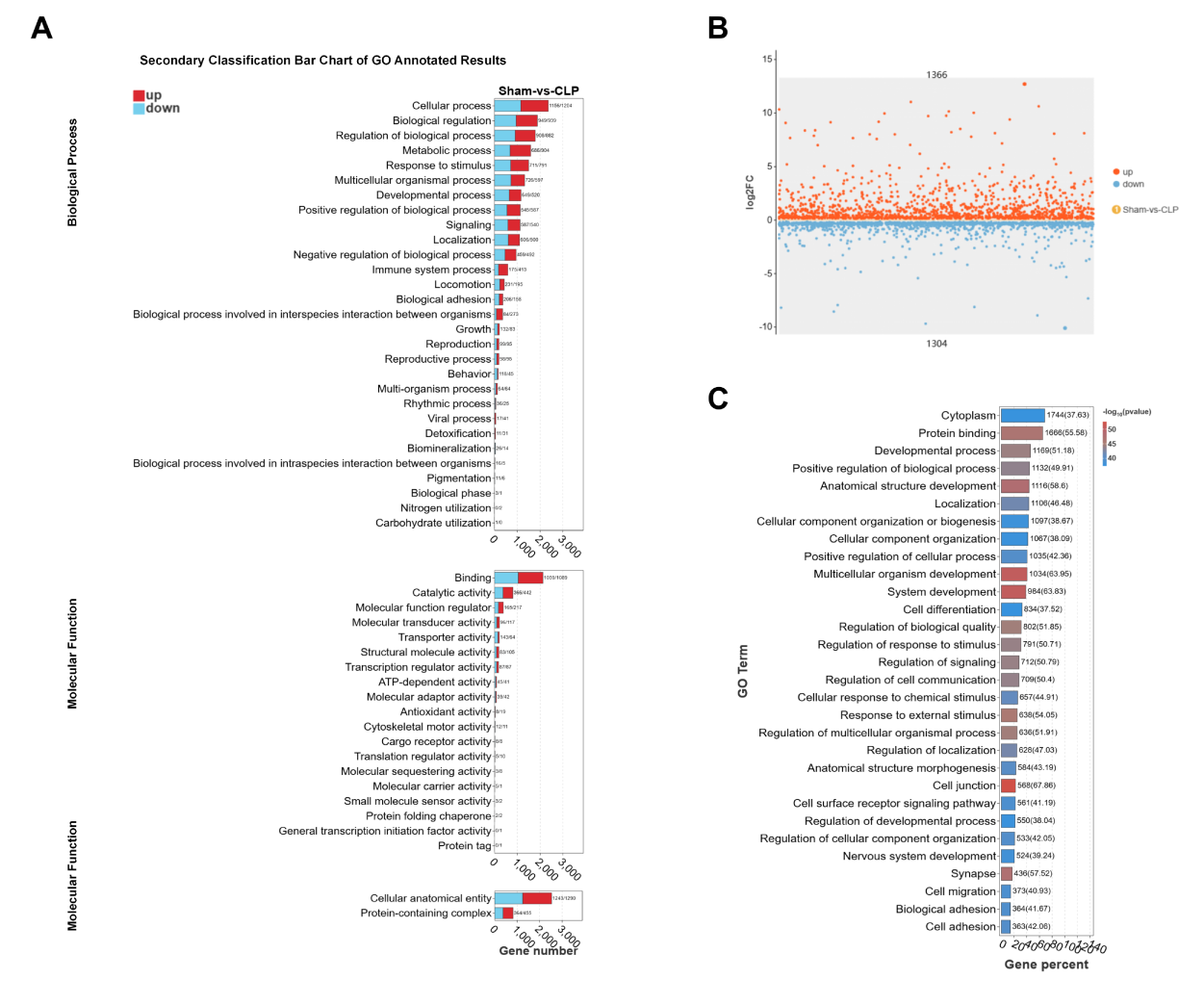
**

**Fig S1. GO enrichment analysis of differentially expressed genes (DEGs) in the thoracic spinal cord from Sham versus CLP-operated mice**

A. Stacked bar charts showing the top-ranked GO definition enrichments assigned from the upregulated and downregulated DEGs in (A) using biological process, molecular function and cellular component definitions. The horizontal axis denotes the number of DEGs associated with each GO definition.

B. Scatter plot visualizing 1366 significantly upregulated (red) and 1304 downregulated (blue) DEGs identified between Sham and CLP group mice. The vertical axis indicates log2(Fold Change).

C. Stacked bar charts showing the top 30 enriched GO pathways. The horizontal axis denotes the proportion of DEGs in each cluster, while the vertical axis indicates the enriched GO terms.

**
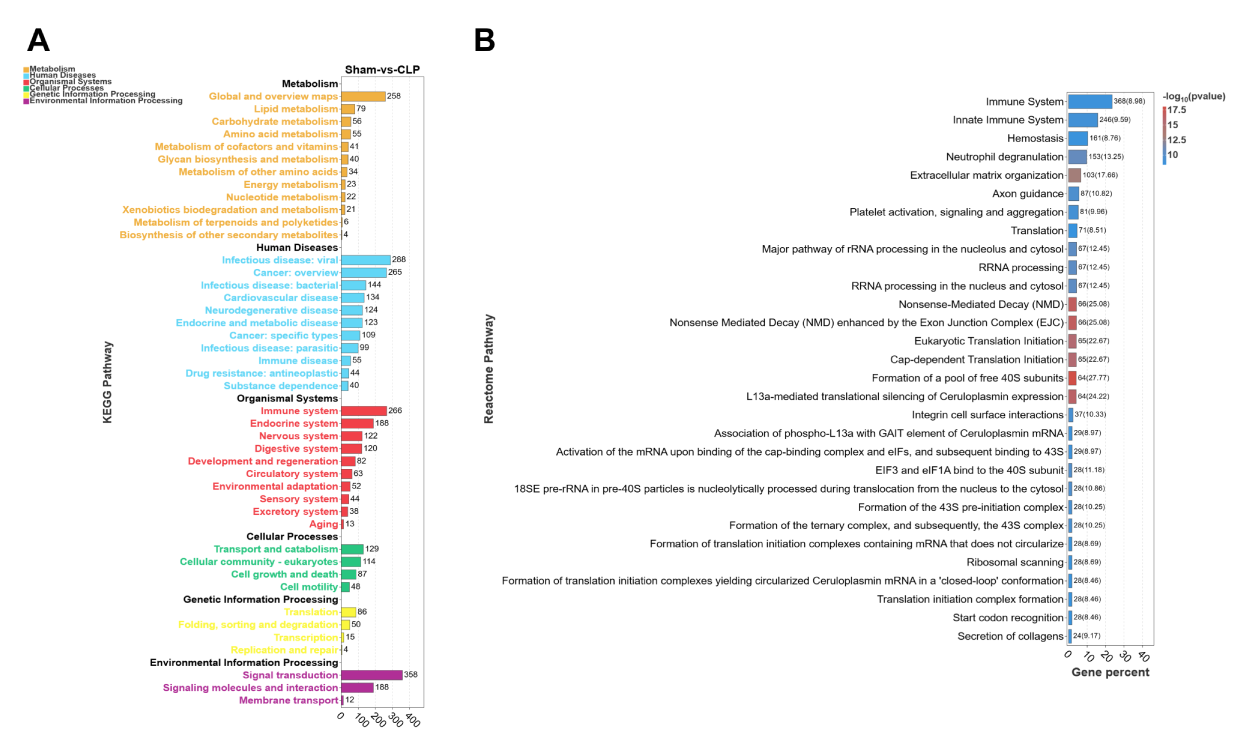
**

**Fig S2. KEGG and Reactome enrichment analysis of DEGs in the thoracic spinal cord from Sham versus CLP-operated mice**

A. The enrichment of DEGs in major KEGG process classifications. The horizontal axis denotes the number of associated DEGs in each pathway.

B. The top 30 most significantly enrichment Reactome terms associated with the DEGs. The horizontal axis denotes the percentage of DEGs with vertical assignments of terms ranked according to p-values.


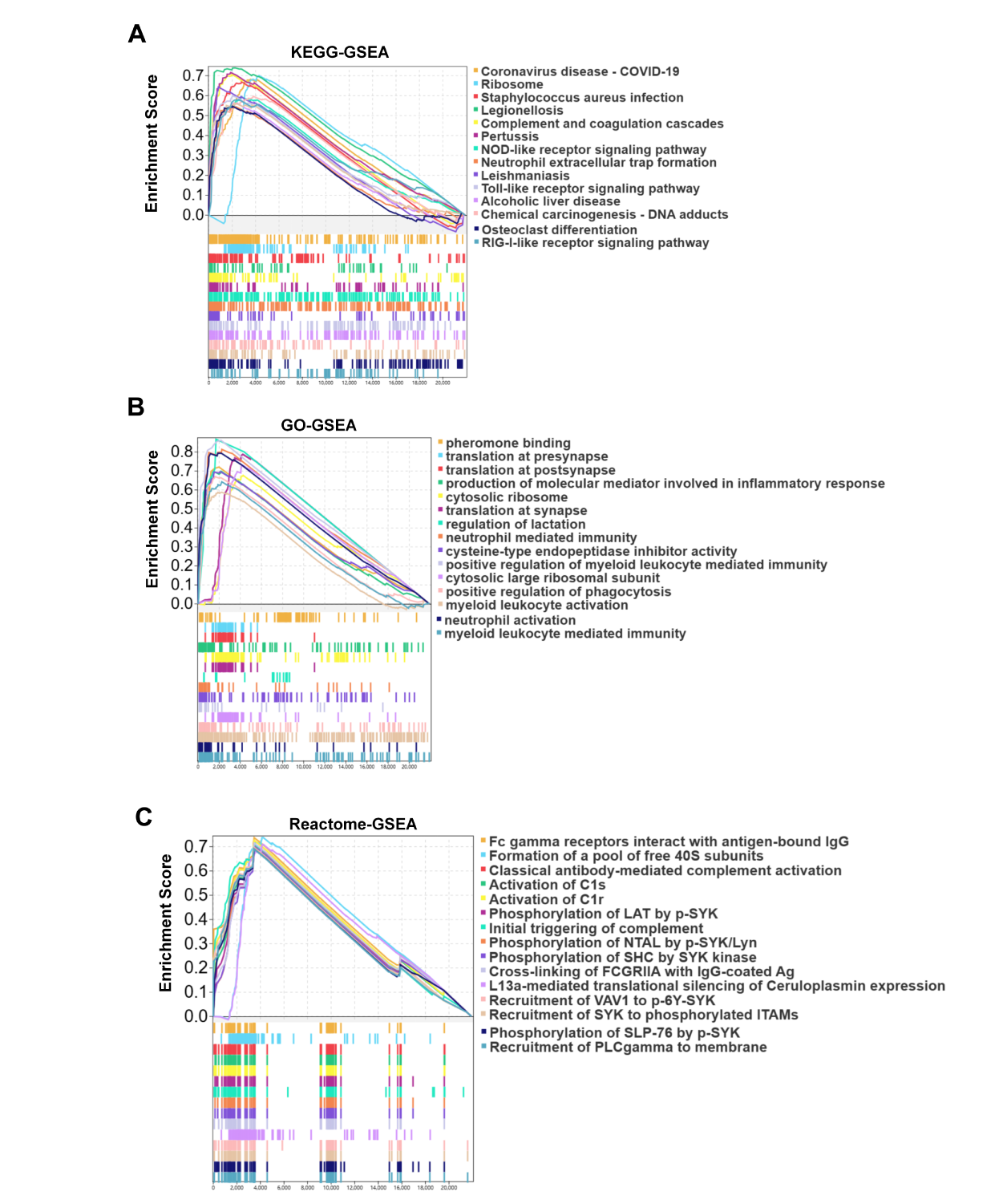


**Fig S3. GSEA enrichment analysis of DEGs against GO, KEGG and Reactome terms**

A-C. Composite GSEA enrichment plots showing top pathways associated with GO (A), KEGG (B) and Reactome (C) terms (top). The y axis represents ranking metric values and the x axis represents the rank for all genes. For each curve, the genes appearing before or at the peak are considered core enrichment genes. The bottom plot corresponding to the colored legend shows the distribution of genes associated with each signature.

**
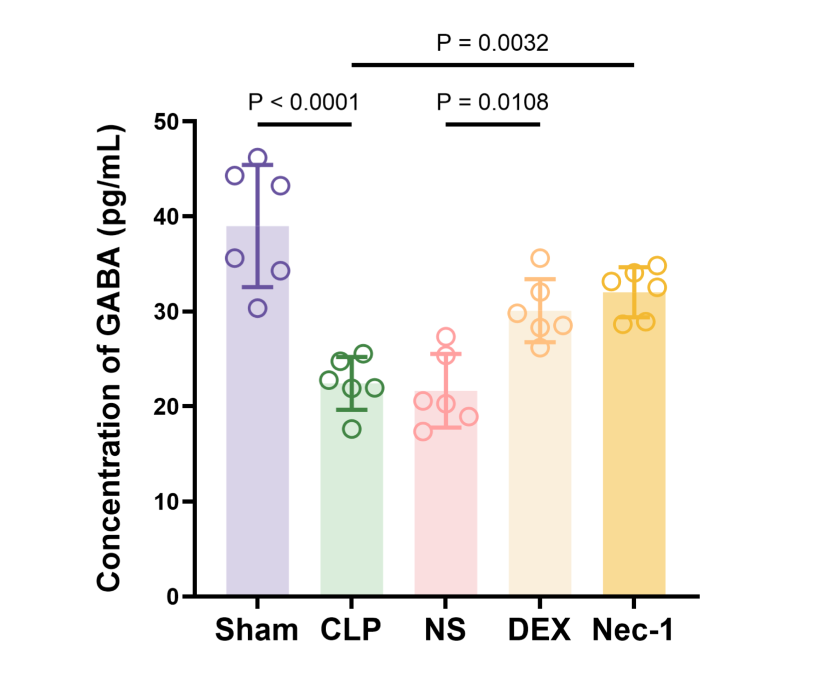
**

**Fig S4. GABA protein concentrations (pg ml^-1^) in the spinal cords of mice from the indicated treatment groups (n=6/group).** Data are shown as the mean ± SD and were compared using one-way ANOVA followed by Tukey's multiple comparisons test.

**
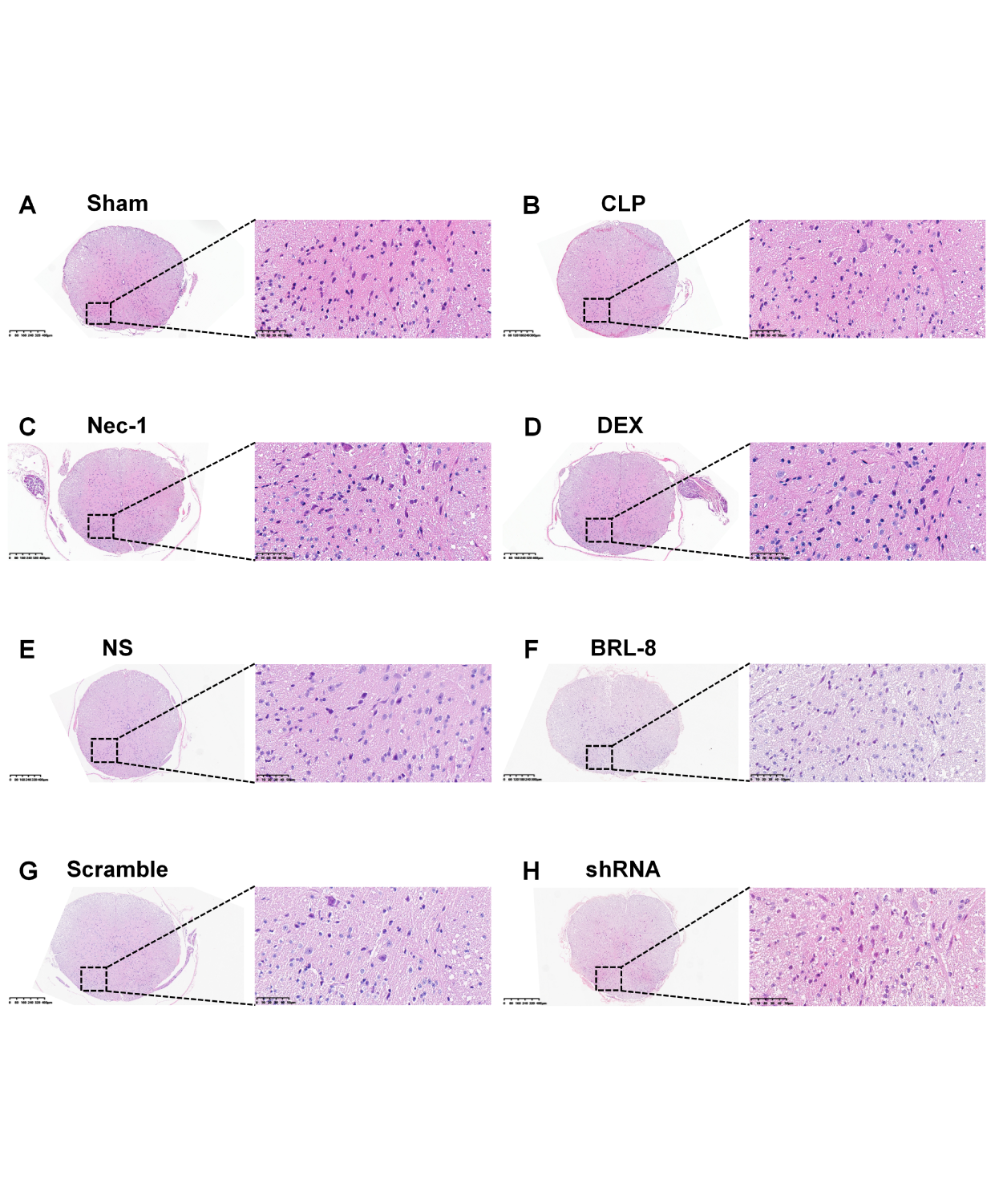
**

**Fig S5. Histological assessment of spinal cord tissues**

Representative transverse sections of hematoxylin and eosin-stained spinal cords from the Sham, CLP, Nec-1, DEX, NS, BRL-8, Scramble and shRNA treatment groups (A-I). Scale bars: 300 μm (main image) and 50 μm (inset).

**
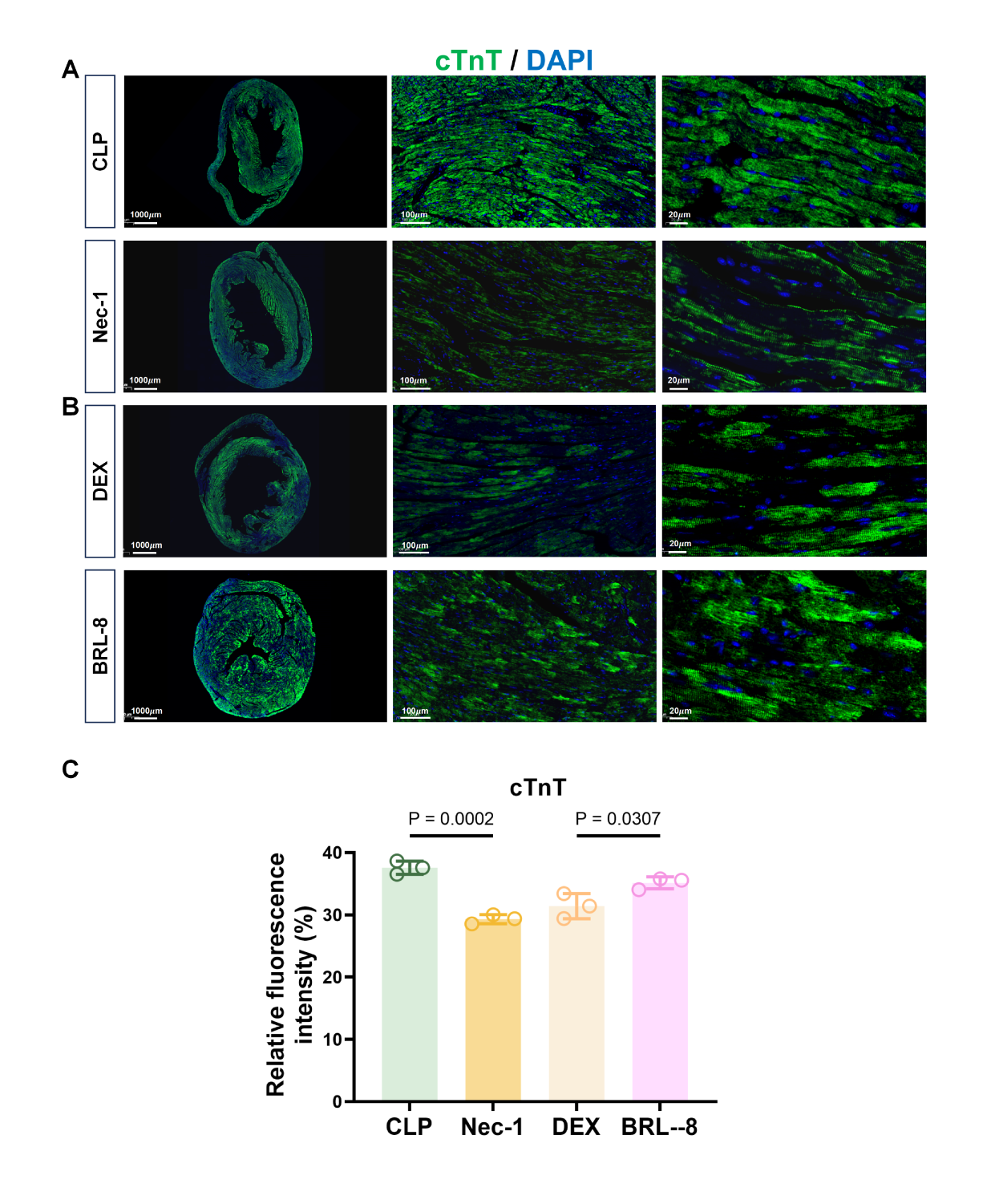
**

**Fig S6. Assessment of cTnT expression in myocardium**

1. B. Representative images of immunofluorescence staining against cTnT (green) and DAPI counterstaining (blue) in heart sections from CLP, Nec-1, DEX and BRL-8 group mice (A and B). Increasing magnification from left to right (scale bars: 1000 μm, 100 μm, 20 μm). Relative cTnT protein expression in heart tissues from (A and B) measured as fluorescence intensity (C) (n = 3/mice per group). Data are shown as the mean ± SD of three independent experiments and were compared using one‐way ANOVA followed by Tukey's multiple comparisons test.

**
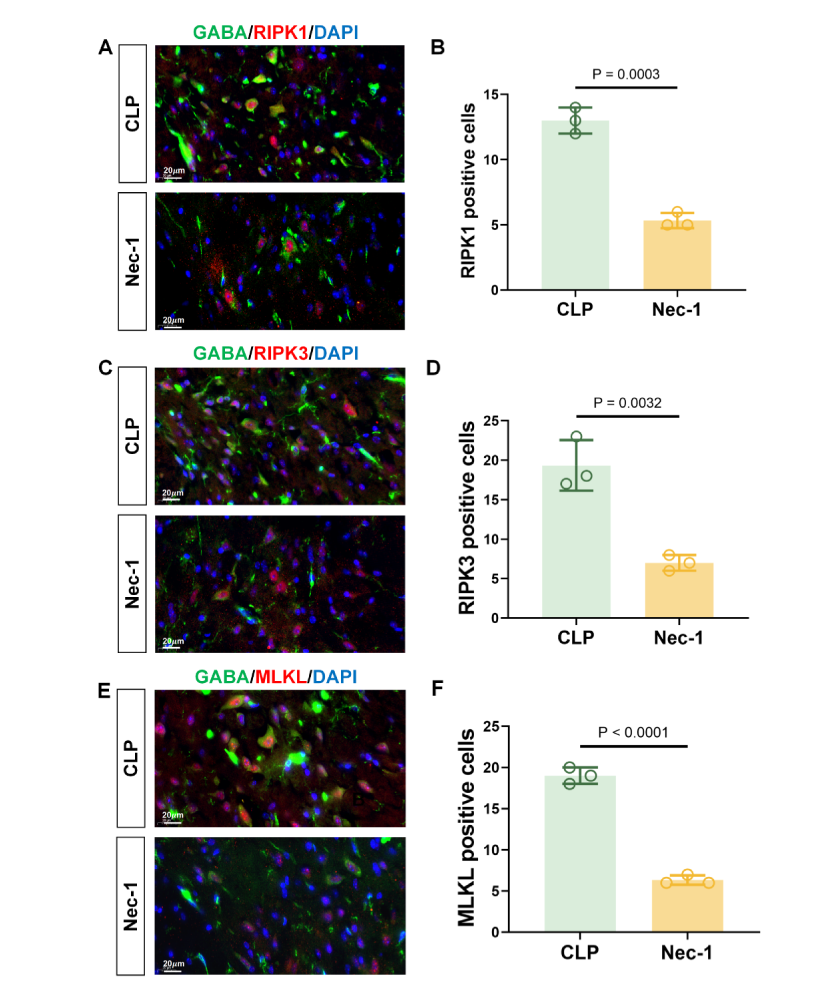
**

**Fig S7. The effect of Nec-1 on neuronal necroptosis in the spinal cord of septic mice**

A-F. Representative epifluorescence images of immunostaining against GABA (green) in combination with RIPK1 (A), RIPK3 (C) or MLKL (E) (red) in thoracic spinal cord sections from CLP and Nec-1 treatment groups. Scale bar: 20 μm. Relative RIPK1 (B), RIPK3 (D) or MLKL (F) levels estimated from fluorescence intensity measurements (n = 3/ sections group). Data are shown as the mean ± SD of three independent experiments and were compared using two‐tailed, unpaired Student's t‐test (B, D and F).

**
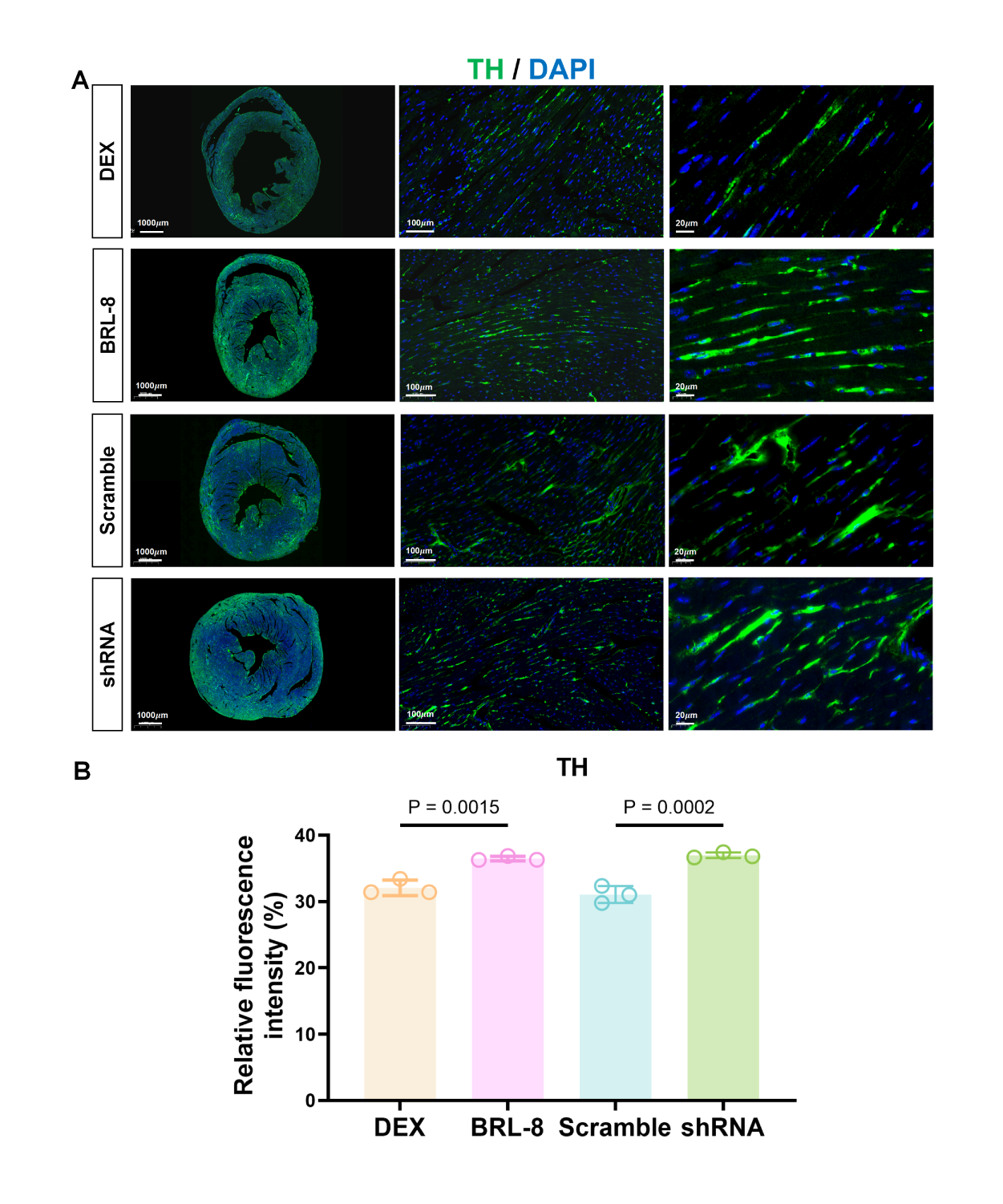
**

**Fig S8. The effects of α2A-AR manipulation on sympathetic excitation in septic mice**

A, B. Representative epifluorescence images of immunostaining against TH (green) in combination with nuclear counterstaining with DAPI (blue) in heart sections from mice in the DEX, BRL-8, Scramble and shRNA treatment groups. Increasing magnification from left to right (scale bars: 1000 μm, 100 μm, 20 μm) (A). Relative TH levels estimated from fluorescence intensity measurements (n = 3/sections group) (B). Data are shown as the mean ± SD of three independent experiments and were compared using one‐way ANOVA followed by Tukey's multiple comparisons test.

**
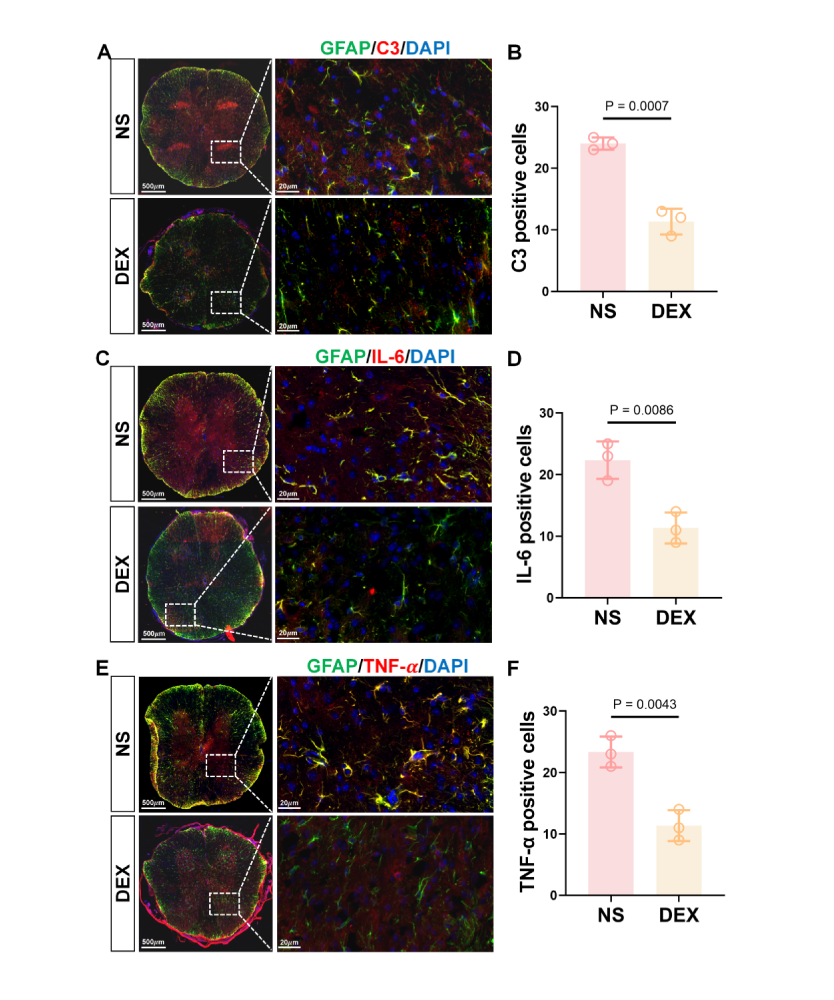
**

**Fig S9. The effect of Dexmedetomidine on spinal cord inflammation in septic mice**

A-F. Representative epifluorescence images of immunostaining against GFAP (green) in combination with C3 (A), IL-6 (C) or TNF-α (E) (red) in thoracic spinal cord sections from control CLP (NS) and DEX treatment groups. Scale bars: 500 μm (left) and 20 μm (right). Relative percentages of cells positive for C3 (B), IL-6 (D) or TNF-α (F) (n = 3/sections group). Data are shown as the mean ± SD of three independent experiments and were compared using two‐tailed, unpaired Student's t‐test (B, D and F).

**Fig S10. Myocarditis responses and cardiac function in septic mice.**

A-D. Representative hematoxylin and eosin-stained sections of heart from mice in the NS (A), DEX (B), Nec-1 (C) and BRL-8 (D) treatment groups. Scale bars: 300 μm (left) and 50 μm (right).

E-H. Cardiac function metrics comparing the DEX and BRL-8 treatment groups. EF (E), FS (F), LVIDd (G), and LVIDs (H) (n = 6 mice/group). I-L. Cardiac function metrics comparing the CLP, DEX (i.p.) and Nec-1(i.p.) treatment groups. EF (I), FS (J), LVIDd (K), and LVIDs (L) (n = 6 mice/group). Data are shown as mean ± SD and were compared using two‐tailed, unpaired Student's t‐test (E-L).

**
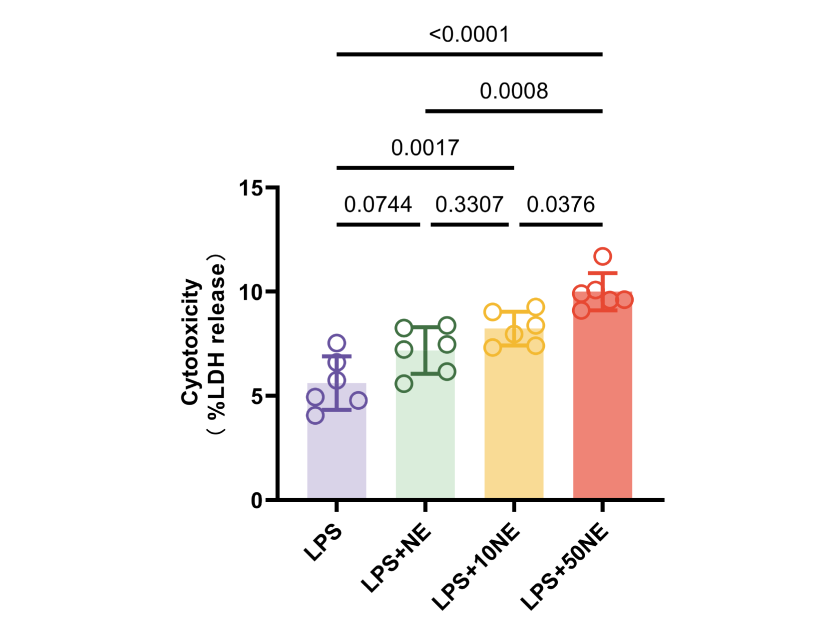
**

**Fig S11. The level of LDH released by cells**

AC16 cells were treated with LPS alone or in combination with 1, 5, or 50 pg mL^-1^ NE for 12h. Cell damage quantitated by LDH assays (n=6 wells/group). Data are shown as the mean ± SD of six independent experiments and were compared using one‐way ANOVA followed by Tukey's multiple comparisons test.

**Table S1. Non-standard Abbreviations and Acronyms**

| **Acronyms** | **Nonstandard abbreviations** |
| --- | --- |
| α2A-AR | Andrenergic receptor alpha-2 |
| BRL-8 | BRL-44408 maleate (an α2A-adrenoceptor antagonist) |
| CCK-8 | Cell Counting Kit 8 assay |
| CNS | Central nervous system |
| CLP | Cecal ligation and puncture |
| cTnT | Cardiac troponin T |
| C3 | Complement3 |
| DEX | Dexmedetomidine |
| EF | Ejection fraction |
| FS | Fractional shortening |
| GABA | γ-aminobutyric acid |
| GFAP | Glial fibrillary acidic protein |
| HE | Hematoxylin and eosin staining |
| IL-6 | Interleukin 6 |
| LV | Left ventricle |
| LVIDd | Left Ventricular Internal Diameter at End-diastole |
| LVIDs | Left Ventricular Internal Diameter at End-systole |
| MLKL | Mixed Lineage Kinase Domain-Like |
| NE | Norepinephrine |
| Nec-1 | Necrostatin-1 |
| PRV | Pseudorabies Virus |
| RIPK1 | Receptor-Interacting Protein Kinase 1 |
| RIPK3 | Receptor-Interacting Protein Kinase 3 |
| RT | Room temperature |
| SCIM | Sepsis-induced cardiomyopathy |
| TNF-α | Tumor Necrosis Factor α |
